# LEM Characterization of Synthetic Jet Actuators Driven by Piezoelectric Element: A Review

**DOI:** 10.3390/s17061216

**Published:** 2017-05-26

**Authors:** Matteo Chiatto, Francesco Capuano, Gennaro Coppola, Luigi de Luca

**Affiliations:** Department of Industrial Engineering, Aerospace Sector, Universitá degli Studi di Napoli “Federico II”, p.le Tecchio 80, 80125 Naples, Italy; francesco.capuano@unina.it (F.C.); gcoppola@unina.it (G.C.); deluca@unina.it (L.d.L.)

**Keywords:** Synthetic Jets (SJ), Lumped Element Model (LEM), piezo-driven actuators, flow control

## Abstract

In the last decades, Synthetic jet actuators have gained much interest among the flow control techniques due to their short response time, high jet velocity and absence of traditional piping, which matches the requirements of reduced size and low weight. A synthetic jet is generated by the diaphragm oscillation (generally driven by a piezoelectric element) in a relatively small cavity, producing periodic cavity pressure variations associated with cavity volume changes. The pressured air exhausts through an orifice, converting diaphragm electrodynamic energy into jet kinetic energy. This review paper considers the development of various Lumped-Element Models (LEMs) as practical tools to design and manufacture the actuators. LEMs can quickly predict device performances such as the frequency response in terms of diaphragm displacement, cavity pressure and jet velocity, as well as the efficiency of energy conversion of input Joule power into useful kinetic power of air jet. The actuator performance is also analyzed by varying typical geometric parameters such as cavity height and orifice diameter and length, through a suited dimensionless form of the governing equations. A comprehensive and detailed physical modeling aimed to evaluate the device efficiency is introduced, shedding light on the different stages involved in the process. Overall, the influence of the coupling degree of the two oscillators, the diaphragm and the Helmholtz frequency, on the device performance is discussed throughout the paper.

## 1. Introduction

It has been many years since Synthetic Jet (SJ) actuators have been used for active flow control, particularly for aerospace applications. These devices are able to manipulate the flow, to modify its structure and allow a favorable variation of the aerodynamic forces on aircrafts (Glezer and Amitay [1], Cattafesta and Sheplak [2], Smith and Glezer [3]). Their application field is extremely wide, including flow control (e.g. Glezer [4] and, more recently, Van Buren et al. [5]), mixing enhancement (Wang and Menon [6], Tamburello and Amitay [7]), heat transfer from small surfaces (Pavlova and Amitay [8], Chaudari et al. [9]), spray vectoring (Pavlova et al. [10], Marchitto et al. [11]), micropropulsion (Finley and Mohseni [12], Parviz et al. [13]), active control for MAV (Otani et al. [14]), and many others.

**Figure 1:**
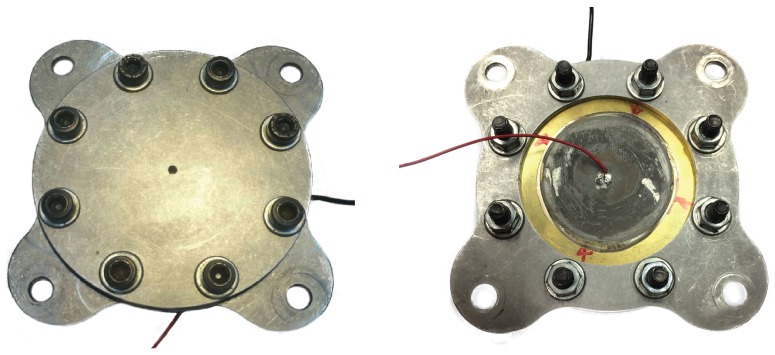
Typical Synthetic Jet device driven by a piezoelectric element. (**left**) top view with orifice; (**right**) bottom view with metallic shim and piezodisk.

**Figure 2:**
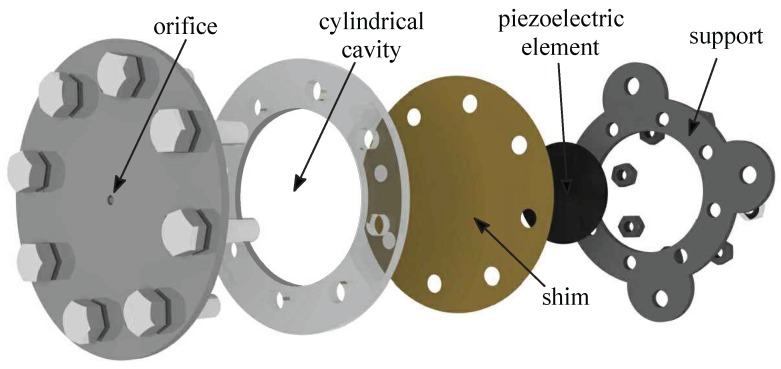
Exploded view of Synthetic Jet (SJ) actuator.

As depicted in Figure 1, a SJ is an electromechanical device consisting of a relatively small cavity, which is sealed from one side by an elastic vibrating diaphragm, and from the other one it is linked to the external environment through a slot or an orifice. This paper will refer to nominally axisymmetric devices only, with round orifice. An exploded view of a typical actuator is reported in Figure 2; the assembly has been designed to fully exploit modularity, allowing independent variations of diameter and height of both the cavity and the orifice. The oscillation of the diaphragm (wall), which is generally constituted by a thin metallic shim on which a piezo-ceramic element is glued, produces periodic cavity volume changes, with corresponding pressure variations, that cause subsequent alternation of ejection and suction phases of fluid across the orifice. During the expulsion phase a vortex ring forms near the orifice exit which, under favorable operating conditions, convects downstream by its self-induced velocity towards the far field. A few cycles are required for the formation of a train of vortex rings that interact with each other and break up due to the viscous dissipation, eventually “synthesizing” a turbulent jet always directed downstream. In more detail, during the suction phase, a stagnation point is formed, located about one orifice diameter away from the orifice itself. This point which separates the near field region, where the flow is directed towards the cavity, from the far field where the jet is established. A major characteristic of this jet is that the average mass flow rate during an operation cycle is null, whilst a non-zero average momentum rate is produced. Furthermore, its generation does not require any continuous fluid supply, because it is synthesized directly from the surrounding fluid, see, e.g., Smith and Glezer [15] and Cater and Soria [16].

Synthetic jets have been extensively studied both from experimental and numerical points of view. Hot-wire Anemometry, Laser Doppler Velocimetry (LDV) and Particle Image Velocimetry (PIV) are only some of the several measurement techniques applied to determine the flow field generated by these devices, see Mohseni and Mittal [17]. On the other hand, many Computational Fluid Dynamics (CFD) simulations have been conducted, ranging from the first Reynolds Averaged Navier-Stokes (RANS) computations to more recent Large Eddy Simulations (LES) and Direct Numerical Simulations (DNS), with the aim to achieve further details of the flow fields inside the cavity as well as near the orifice, both in quiescent and cross-flow conditions (e.g., Rumsey et al. [18], Dandois et al. [19], Lardeau and Leschziner [20]). Another very useful tool, employed for design and manufacturing purposes, consists in the definition of a low-order model, able to predict the dynamic response of the actuator in a relatively quick way and with reasonable fidelity and accuracy.

Within this latter class of modeling, Lumped Element Models represent a very practical tool to obtain the time variation of all thermodynamic variables inside the cavity as well as the jet velocity issuing from the orifice, as functions of the operating frequency. One of the earliest lumped modeling applications to synthetic jets has been presented by McCormick [21], who developed a simplified electro-acoustic model predicting the velocity performance of a SJ actuator driven by a loudspeaker acoustic forcing. The extension of this model to a piezoelectric-driven device (constituted of a thin piezo-ceramic disk glued on a metallic shim) has been carried out by Prasad [22] and further studied by Gallas et al. [23], who modeled the individual components of the actuator as elements of an equivalent electric circuit, including a very detailed description of the behavior of the composite oscillating diaphragm. The model was validated against experimental measurements of the orifice exit velocity. Following the electroacoustic approach, many other lumped models have been realized, which take into account more and more effects, such as details about the piezoelectric element (Prasad et al. [24], Persoons [25]), minor losses (Tang and Zhong [26]), electromagnetic driver (Agashe et al. [27], Sawant at al. [28]) and double cavity (Luo et al. [29], Arunajatesan [30]). Later on, a physical model directly based on fluid-dynamics equations has been presented by Sharma [31], who considered the oscillating wall as a single-degree-of-freedom mechanical system, while the cavity-orifice arrangement is basically described by suited forms of continuity and Bernoulli’s unsteady equations. This last model inspired the work of de Luca et al. [32], who provided additional analytical and numerical insights on the frequency response of SJ actuators driven by piezoelectric thin elements (among others, the prediction of the coupled resonance frequencies and the conditions to maximize the peak velocity). To sum up, lumped element models can be clustered in two categories: the former based on an equivalent electrical circuit; the latter fully based on fluid-dynamics equations. In both cases, the device is considered as a coupled mechanical-acoustic resonator with two degrees of freedom, exhibiting two resonance frequencies near the uncoupled Helmholtz and diaphragm structural resonance frequencies.

The aim of this work is to present a comprehensive review covering the development and the evolution of LEM modeling for the design and the performance evaluation of synthetic jet actuators. The subject is basically developed as follows.

Section 2 deals with the description of different lumped models that have been developed to characterize SJ devices. A careful description of these models appears to be mandatory to emphasize their strength but also their limitations. Therefore, the goal of this part is to present the basic assumptions on which the models are based, and the governing equations. Their dimensionless form is also developed, and the Strouhal and the coupling factor are introduced.

Section 3 is dedicated to comparing the results produced by two basic LEM approaches, one fully based on the fluid dynamics equations, and the other obtained within the so-called equivalent electric circuit framework. Such a comparison is also corroborated by comparisons of numerical results with experimental measurements.

Section 4 inspects the major device performances (basically the frequency response in terms of jet velocity), with particular attention to the role played by the parameters that influence the oscillators coupling, the supply voltage, and the effective orifice length.

Section 5 contains the analysis of SJ actuators efficiency, based on a physical model directly related to the energy equations of the two coupled oscillators, and it is followed by Section 6 where some concluding remarks are drawn.

## 2. Lumped Element Models

A lumped element model is a low order model which, under certain assumptions, allows to describe the behavior of a physical system through a finite number of components (lumped elements) properly connected to each other. In general, the typical size of each component is small relative to a characteristic wavelength. This simplification decouples temporal and spatial variations, since the component is considered as being concentrated at a nodal point, and thus the partial differential equations of the distributed system are reduced to a set of coupled ordinary differential equations in time. The lumped modeling is a practical tool for design and manufacturing intents, and provides the dynamic response of a complex system in a relatively short computation time with a sufficient level of accuracy. Within the present context, with reference to the cavity flow which undergoes alternate compression and expansion phases due to the wall oscillations, the internal pressure can be considered uniform at any given time instant if the wavelength of the pressure oscillations is much larger than a typical dimension of the actuator cavity, Sharma [31]. In other terms, the basic assumption is that the crossing time of an acoustic perturbation generated by the elastic diaphragm, traveling from the diaphragm to the opposite (rigid) cavity walls, is much smaller than the actuation period; in this case, the acoustic perturbation generated at a (moving) boundary is instantaneously imposed to the whole control volume (namely, the cavity). The lumped assumption may of course become invalid at very high frequencies, de Luca et al. (2016) [33].

Most previous LEM contributions on SJ devices are based on the electric circuits analogy (McCormick [21], Prasad et al. [22], Gallas et al. [23] and Persoons [25]). Due to their intrinsic nature, these models yield the system stationary (periodic) solution, and focus their attention on the evaluation of the impedances of the various circuit components. On the contrary, fluid dynamics based LEMs generally result in an initial value problem of ordinary differential equations and the solution is obtained numerically by integrating in time the governing equations with, for instance, Runge-Kutta type methods (Sharma [31] and de Luca et al. [32]); however, it will be shown in Section 2.2 how the stationary solution can be directly obtained by enforcing analytical *modal* solutions to the problem. In this paper the fluid dynamics approach is mostly discussed; it will be presented hereafter through the formulations described by Sharma [31] and de Luca et al. [32]; then, once defined the two-coupled-oscillator mechanical layout, the mass-inductance electro-mechanical analogy is applied to obtain the equivalent electric circuit and the relevant impedances.

### 2.1. Fluid Dynamic LEM

A model directly based on fluid mechanics equations was presented originally by Sharma [31] and extended by de Luca et al. [32] later on. The model is able to predict cavity pressure fluctuations, flow velocity at the orifice exit section, average displacement and velocity of diaphragm, as well as phase-lag relationships between the different variables. As anticipated in the previous paragraph, the three basic elements of the actuator are modeled: the oscillating diaphragm, the cavity and the orifice.

**Figure 3:**
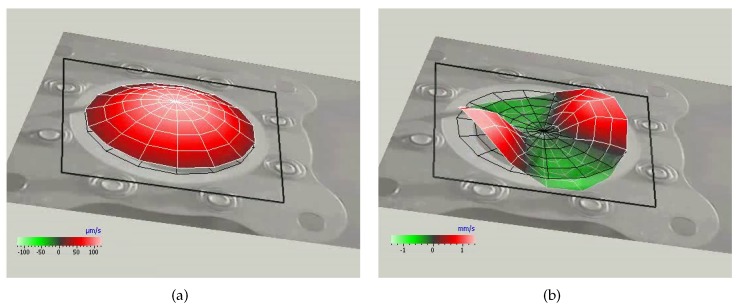
Aluminum shim diaphragm deflections detected by laser vibrometer. (**a**) fundamental mode (0,1) for actuation frequency f=1555 Hz; (**b**) mode (2,2) for f=5370 Hz. $V_{ac}=6$ V. Courtesy of University of Naples.

The dynamics of the diaphragm is described through the equation of motion of a one-degree-of-freedom forced-damped spring-mass system:

(1) $${\ddot{x}}_{w}+2\zeta_{w}\omega_{w}{\dot{x}}_{w}+\omega_{w}^{2}x_{w}=\omega_{w}^{2}\Delta x_{w}\sin\omega t-\frac{p_{i}A_{w}}{m_{wt}}$$

where $x_{w}\left(t\right)$ is the diaphragm (average) displacement at a generic time instant *t*, $\zeta_{w}$ is the actual damping ratio of the diaphragm, $\omega_{w}$ its natural frequency, $\Delta x_{w}$ is the average linear diaphragm displacement due to the application of a certain voltage to the piezo-element, ω is the operating frequency, and the dot superscript stands for time derivative. Furthermore, $p_{i}$ represents the cavity (internal) differential pressure, $A_{w}$ is the diaphragm surface area and $m_{wt}$ is the diaphragm total mass, including shim, piezo-element and air added mass. The structural frequency of the composite diaphragm is defined as:

(2) $$\omega_{w}=\sqrt{\frac{k_{w}}{m_{wt}}}$$

which represents the uncoupled (first mode) natural frequency of the structural oscillator, where $k_{w}$ is the diaphragm equivalent spring stiffness. This latter is evaluated as:

(3) $$k_{w}=m_{w}{\left(2\pi {\tilde{f}}_{w}\right)}^{2}$$

where ${\tilde{f}}_{w}$ is the frequency of the principal vibration mode of a rigidly clamped disk, assumed equal to the first fundamental mode of the shim only (Rathnasingham and Breuer [34] and Kinsler et al. [35]), that is the structural element actually clamped, while $m_{w}$ is the diaphragm mass including both shim and piezo-ceramic disk. Higher modes also can be excited at relatively high frequencies, as depicted in Figure 3, where the diaphragm deflection detected by a Polytec laser scanning vibrometer PSV400-H4 is reported for operation frequencies *f* of 1555 Hz and 5370 Hz, respectively, and very low voltage, $V_{ac}=6$ V. The device has an aluminum shim with a diameter of 42 mm, while the chamber is 3 mm in height. Note that the presence of higher-order structural modes is not desirable because the distorted shim shape vibrating at natural frequencies higher than its fundamental one produces little net displacement of the surrounding air.

The electro-dynamic force (applied to the diaphragm by the piezoelectric element) is modeled as $F=F_{o}\sin\omega t$, with $F_{o}$ being the force magnitude:

(4) $$F_{o}=\frac{k_{w}d_{a}V_{ac}}{A_{w}}=k_{w}\Delta x_{w}$$

where the average linear displacement $\Delta x_{w}$ is expressed as the cavity volume variation $\Delta V=d_{a}V_{ac}$ divided by the wall area, and $V_{ac}$ represents the applied voltage. It is important to note that, despite what is shown by Equation (4), the dependence of the dynamic deflection of the piezo-element on the driving voltage is not linear, but the slope of the curve deflection-voltage decreases with increasing voltage, and it is a function of the operating frequency, as investigated by Krishnan and Mohseni [36]. This behavior of the piezo-materials influences the performances of the actuators, as will be shown in Section 4.2. The effective acoustic piezoelectric coefficient $d_{a}$ is defined as the ratio of the volume variation to the applied voltage, when the driving differential pressure across the piezo-element is null, Prasad [22],

(5) $$d_{a}=\frac{\Delta V}{V_{ac}}{|}_{p=0}$$

The determination of $d_{a}$ could not be trivial since it requires the knowledge of the transverse displacement distribution of the composite diaphragm, as outlined by Prasad [22] and Prasad et al. [24]; de Luca et al. [32] observed that a more convenient procedure consists in determining the acoustic compliance of the diaphragm $C_{w}$ as:

(6) $$C_{w}=\frac{\Delta V}{p}{|}_{V_{ac}=0}$$

and to relate it to $d_{a}$ through the electroacoustic transduction coefficient $\Phi_{a}$:

(7) $$d_{a}=C_{w}\Phi_{a}$$

The acoustic compliance is then determined from piezo-ceramic properties:

(8) $$C_{w}=\frac{\pi d_{pc}^{6}\left(1-\nu_{pc}^{2}\right)}{1024E_{pc}th_{pc}^{3}}$$

with $th_{pc}$, $d_{pc}$, $E_{pc}$, and $\nu_{pc}$ being the thickness, the diameter, Young’s modulus, and Poisson’s ratio of the piezo-ceramic respectively. From their physical definitions, the relationship between the stiffness and the compliance is easily obtained:

(9) $$C_{w}=\frac{A_{w}^{2}}{k_{w}}$$

Because of the difficulties in determining the electro-mechanical properties of the composite diaphragm, in practical applications the modeling described above considers the electroacoustic transduction coefficient $\Phi_{a}$ as a fitting parameter to be determined by comparisons between numerical and experimental data.

The second equation of the model is represented by the conservation of mass inside the cavity under the assumption of zero-dimensional system. By relating the density and pressure variations by means of an isentropic compression/expansion transformation, the continuity equation can be formulated as:

(10) $$\frac{V_{c}}{\gamma p_{a}}\frac{dp_{i}}{dt}-A_{w}{\dot{x}}_{w}=-A_{o}U$$

where $V_{c}=A_{w}H$ is the cavity volume (with *H* being the cavity height), $A_{o}$ is the orifice area, γ is the specific heat ratio of air, $p_{a}$ is the external ambient pressure and *U* is the instantaneous flow velocity through the orifice, namely the jet velocity.

Finally, the model is completed with the unsteady Bernoulli’s equation, applied between a point inside the cavity, where the flow velocity is practically null and a point just outside the cavity, which represents the location where the pressure matches the unperturbed external ambient value:

(11) $$\ddot{U}+\frac{K}{l_{e}}\left|U\right|\dot{U}+\omega_{H}^{2}U=\frac{A_{w}}{A_{o}}\omega_{H}^{2}{\dot{x}}_{w}$$

in Equation (11), *K* is the head loss coefficient, including the inviscid contribution (equal to unity) due to the kinetic energy recovery at ambient pressure, minor (entrance/exit) losses, and distributed losses due to friction inside the orifice duct. The distance between the two points of application of the Bernoulli’s equation is referred to as the modified (effective) length of the orifice, $l_{e}$. More details and typical values for *K* can be found in Sharma [31] and de Luca et al. [32]. The Helmholtz frequency is defined as:

(12) $$\omega_{H}=\sqrt{\frac{\gamma A_{o}^{2}p_{a}/V_{c}}{\rho_{a}l_{e}A_{o}}}=\sqrt{\frac{k_{a}}{m_{a}}}$$

with $k_{a}$ and $m_{a}$ being the equivalent stiffness of the air inside the cavity and the effective mass of the air at the orifice.

By taking the time derivative of Equation (1), and eliminating the pressure derivative by means of Equation (10), de Luca et al. (2016) [33], obtained:

(13) $${\ddot{V}}_{w}+2\zeta_{w}\omega_{w}{\dot{V}}_{w}+\left(\omega_{w}^{2}+\omega_{wc}^{2}\right)V_{w}=\frac{A_{o}}{A_{w}}\omega_{wc}^{2}U+\left(\omega \Delta x_{w}\right)\omega_{w}^{2}\cos\omega t$$

which has to be coupled with Equation (11). $V_{w}={\dot{x}}_{w}$ denotes the diaphragm velocity. Following Sharma [31], the frequency $\omega_{wc}$ represents the natural frequency of the pneumatic spring made of the air enclosed within the cavity volume, $V_{c}$, and the oscillating diaphragm of mass $m_{w}$:

(14) $$\omega_{wc}=\sqrt{\frac{\gamma A_{w}^{2}p_{a}/V_{c}}{m_{wt}}}=\sqrt{\frac{\gamma A_{w}p_{a}}{m_{wt}H}}$$

To sum up, the actuator behavior is modeled by the dynamics of two mutually coupled oscillators: the first one, describing the diaphragm motion, Equation (13), is characterized by the uncoupled natural frequency $\omega_{w}$, while the second one, the acoustic oscillator, Equation (11), describing the dynamics of the mass of air at the orifice, $m_{a}$, through its velocity *U*, is characterized by its natural frequency $\omega_{H}$. An external forcing due to the supply power also acts on the diaphragm dynamics.

Making the further assumption of absence of damping effects (the practical validity of such an assumption has been discussed deeply in previous papers), for temporal behavior of the free oscillations $∼e^{j\omega_{n}t}$, a closed-form analytical evaluation of the natural coupled (or modified) frequencies is obtained:

(15) $$\omega_{n}^{2}\equiv \omega_{1,2}^{2}=\frac{\left(\omega_{w}^{2}+\omega_{wc}^{2}+\omega_{H}^{2}\right)\pm \sqrt{{\left(\omega_{w}^{2}+\omega_{wc}^{2}+\omega_{H}^{2}\right)}^{2}-4\omega_{H}^{2}\omega_{w}^{2}}}{2}$$

The natural coupled frequencies in the totally undamped case are often also expressed conveniently in Hz, namely:

(16) $$f_{1,2}=\frac{\omega_{1,2}}{2\pi }$$

Despite the seemingly strong simplification, predictions based on the previous relationship are in several circumstances in very good agreement with data obtained by experimental measurements, de Luca et al. [32]. In some applications, a desirable operating condition is represented by a relatively high plateau of fluid ejection velocity over a rather wide range of frequencies. This condition can be attained by designing the devices so as to have the two frequencies $\omega_{1}$ and $\omega_{2}$ close to each other. In fact, upon inspection of Equation (15), one argues that when $\omega_{w}\ll \omega_{H}$, the distance between the frequencies is a function of the cavity height:

(17) $$\left|\omega_{1}^{2}-\omega_{2}^{2}\right|≅\frac{1}{H/d_{o}}$$

On the other hand, if $\omega_{w}\gg \omega_{H}$, then:

(18) $$\left|\omega_{1}^{2}-\omega_{2}^{2}\right|≅const$$

**Figure 4:**
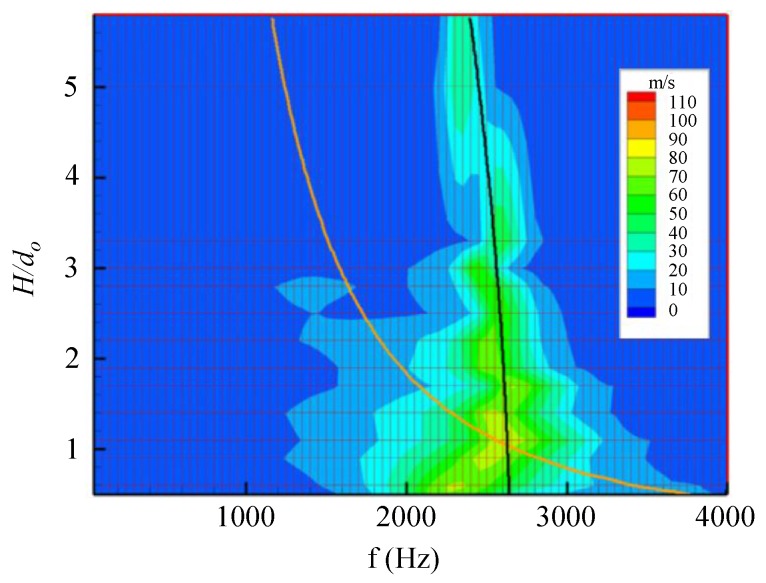
Plan view of device response in peak velocity as a function of the chamber height $H/d_{o}$. Coupled structural frequency (black line) and Helmholtz frequency (orange line) are depicted. Reprinted with permission from [37].

The above findings were obtained experimentally by Gomes et al. [37], who carried out extensive measurements to characterize piezo-driven devices of the same kind of those studied theoretically in this paper. Figure 4 shows the variations of the coupled structural frequency (black line) and of the Helmholtz frequency (orange line) as functions of the dimensionless chamber height.

These results will be further discussed in Section 4 along with comparisons to experimental findings. It is interesting to observe that some experimentalists do not measure the jet velocity at the orifice section, but about one-diameter downstream of the exit, i.e., approximately at the stagnation point (named also saddle point), by means of a rather simple Pitot tube. To understand the correlation between the saddle point velocity and the orifice (centerline) peak velocity, it is convenient to recall some characteristic quantities of the device. Since the jet formation is related to the ability of the vortex ring produced in the ejection phase to escape during the ingestion phase, a parameter characterizing the jet strength is the so called stroke length $\bar{L}$, namely the integral of the spatially averaged velocity at the orifice exit over the cycle ejection phase only:

(19) $$\bar{L}=\int_{0}^{T/2}U\left(t\right)dt$$

where *T* is the actuation period. A proper reference velocity is introduced via the relation $\bar{U}=\bar{L}/T$ or, in other terms, as:

(20) $$\bar{U}=\frac{1}{T}\int_{0}^{T/2}U\left(t\right)dt$$

which is usually refereed to as stroke length velocity. According to classic literature findings (Smith and Glezer (1998) [15] and Smith and Glezer (2002) [3]), the saddle point velocity, $U_{e}$, is roughly ≈1.1 times the stroke length velocity, which in turn, for sinuous time variation of the exit velocity, is related to its peak value by $\bar{U}=U_{\max}/\pi$. Thus, to compare experimental measurements to numerical computations of the peak value $U_{\max}$, the following relationship is used:

(21) $$U_{e}=1.1\frac{U_{\max}}{\pi }$$

The frequency response of SJ actuator is presented very often in terms of saddle point velocity, as will be hereafter reported when discussing overall data of literature.

It is worth to observe that in the fluid dynamic LEM approach the time dependent governing equations constitute an initial value problem to be integrated numerically by means of more or less standard numerical schemes such as Runge-Kutta methods. Details about the numerical procedure are reported in Sharma [31] and de Luca et al. [32]. Here, the numerical simulations are carried out in MATLAB environment with *ode45* routine. Initial conditions of $x_{w}=0$, $V_{w}=0$, $p_{i}=0$ and U=0 are assumed in the computations. It has been observed that the quasi-steady oscillatory solution is generally reached in about 20–30 cycles. Typical values of the electroacoustic transduction coefficient $\Phi_{a}$ that best fit the continuous numerical curves to the velocity measurements range from 40 to 150 Pa/V, depending on the actuator under consideration. Such values are compatible with physical values reported by Gallas et al. [23] and Prasad et al. [24].

### 2.2. Analytical Stationary Solution

It has been already observed that the LEM modeling based on the electric circuit analogy yields basically the periodic stationary solution. The LEM electric analogy will be treated in the next section. Hereafter, an analytical *modal* approach to obtain the stationary solution of the jet velocity will be described, starting from the LEM fluid dynamics equations. Note that, in spite of the presence of the nonlinear damping term in the acoustic oscillator model, Equation (11), the *modal* approach is successful because the non-linearity is represented by the modulus of the unknown *U*. The following development represents an original reformulation of the previous one reported in de Luca et al. (2016) [33], including a specific evaluation of the magnification factor in resonance conditions, as well as some asymptotic estimates.

The dynamic model constituted by Equations (11) and (13) can be conveniently rewritten as

(22) $$\begin{array}{c} \ddot{\left[\begin{array}{c} V_{w}\\ U \end{array}\right]}+\left[\begin{array}{cc} 2\zeta_{w}\omega_{w} & 0\\ 0 & \frac{K}{l_{e}}\left|U\right| \end{array}\right]\dot{\left[\begin{array}{c} V_{w}\\ U \end{array}\right]}+\\ \;\;\;\;\;+\left[\begin{array}{cc} \frac{1}{m_{wt}} & 0\\ 0 & \frac{1}{m_{a}} \end{array}\right]\left[\begin{array}{cc} k_{w}+\frac{\gamma p_{a}}{V_{c}}A_{w}^{2} & -\frac{\gamma p_{a}}{V_{c}}A_{0}A_{w}\\ -\frac{\gamma p_{a}}{V_{c}}A_{0}A_{w} & \frac{\gamma p_{a}}{V_{c}}A_{0}^{2} \end{array}\right]\left[\begin{array}{c} V_{w}\\ U \end{array}\right]=\left[\begin{array}{c} \frac{F_{0}}{m_{wt}}\omega \cos\omega t\\ 0 \end{array}\right] \end{array}$$

**Figure 5:**
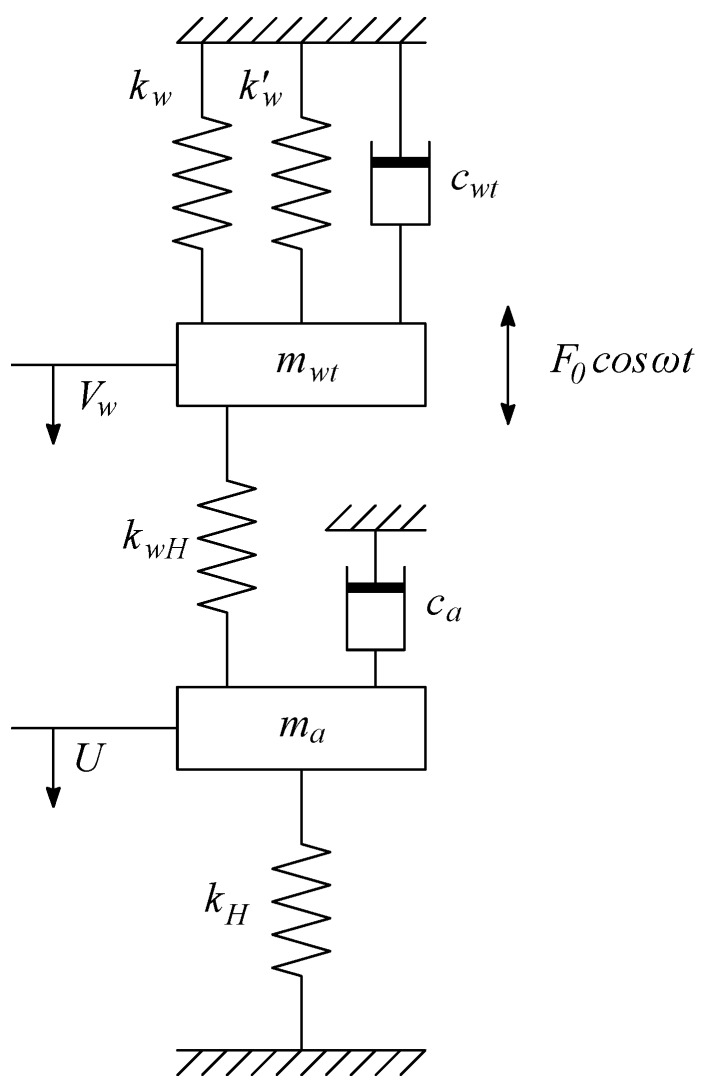
Forced damped spring-mass schematic of SJ actuator.

Equation (22) is representative of a canonical damped spring-mass oscillators system, with the peculiar feature of having a non-linear damping factor. Note also that the *stiffness matrix* is explicitly shown, see Equations (12) and (14). A graphical representation of this system is provided in Figure 5, where $k_{w}^{\prime }=\frac{\gamma p_{a}}{V_{c}}A_{w}^{2}$, $k_{wH}=k_{Hw}=\frac{\gamma p_{a}}{V_{c}}A_{0}A_{w}$, and $k_{H}=\frac{\gamma p_{a}}{V_{c}}A_{0}^{2}$.

Steady-state solutions can be sought by introducing the *normal mode* assumption:

(23) $$\left[\begin{array}{c} V_{w}\\ U \end{array}\right]=\left[\begin{array}{c} {\tilde{V}}_{w}\\ \tilde{U} \end{array}\right]e^{j\omega t}$$

where ${\tilde{V}}_{w}$ and $\tilde{U}$ are complex quantities. Also, $\frac{F_{0}}{m_{wt}}e^{j\omega t}$ is substituted to $\frac{F_{0}}{m_{wt}}\omega \cos\omega t$. It is worth to note that $|\tilde{U}|\equiv U_{\max}$.

The resulting algebraic, non-linear system can be implicitly solved to give the *amplification factor* of the maximum jet velocity with respect to the incompressible velocity, $U_{\mathrm{inc}}=\left(A_{w}/A_{0}\right)\omega \Delta x_{w}$. Upon back-substitution of the undamped (coupled) resonance frequencies $\omega_{1,2}$, Equation (15), one obtains

(24) $$\frac{U_{\max}}{U_{\mathrm{inc}}}=\frac{1}{\sqrt{{\left[2\zeta_{w}\frac{\omega }{\omega_{w}}\delta_{H}+2\zeta_{U}\frac{\omega }{\omega_{H}}\delta_{CF}\right]}^{2}+{\left[\delta_{1}\delta_{2}+4\zeta_{w}\zeta_{U}\frac{\omega }{\omega_{w}}\frac{\omega }{\omega_{H}}\right]}^{2}}}$$

where the known relationship $\omega_{1}\omega_{2}=\omega_{H}\omega_{w}$ has been acknowledged, $\delta_{\left(\cdot \right)}=\left(1-\omega^{2}/\omega_{\left(\cdot \right)}^{2}\right)$, $\delta_{CF}=\left(1+CF-\omega^{2}/\omega_{w}^{2}\right)$, and CF is defined as the *coupling factor* of the two oscillators:

(25) $$CF=\frac{\omega_{wc}^{2}}{\omega_{w}^{2}}$$

Also, $\zeta_{w}$ and $\zeta_{U}=\left(U_{\max}K\right)/\left(2\omega_{H}l_{e}\right)$ are the non-dimensional structural and fluid dynamic (or acoustic) damping factors respectively. Furthermore, observe that the dimensionless frequency $\omega /\omega_{H}$ can be related to the operation Strouhal number, which will be introduced in next Section 2.4. Since the Equation (24) is still implicit in $U_{\max}$, it has to be solved numerically to obtain the analytical stationary solution of the jet velocity as a function of the operating frequency ω. Some of these solutions will be reported in Section 3, when discussing comparisons of LEM results available for the devices tested by de Luca et al. [33].

Various simplified relationships can be derived starting from the basic Equation (24), which can be used to confirm the numerical predictions presented in Section 4. For instance, in the case of uncoupled oscillators (CF=0), $\omega_{1}\equiv \omega_{H}$ and $\omega_{2}\equiv \omega_{w}$, thus Equation (24) becomes:

(26) $${\left.\frac{U_{\max}}{U_{\mathrm{inc}}}\right|}^{CF=0}=\frac{1}{\sqrt{{\left[2\zeta_{w}\frac{\omega }{\omega_{w}}\delta_{H}+2\zeta_{U}\frac{\omega }{\omega_{H}}\delta_{w}\right]}^{2}+{\left[\delta_{H}\delta_{w}+4\zeta_{w}\zeta_{U}\frac{\omega }{\omega_{w}}\frac{\omega }{\omega_{H}}\right]}^{2}}}$$

The amplification factor can be conveniently evaluated in correspondence of the resonance frequencies. In particular, when the system is driven at $\omega =\omega_{w}$, one obtains:

(27) $${\left.\frac{U_{max}}{U_{\mathrm{inc}}}\right|}_{\omega =\omega_{w}}^{CF=0}=\frac{1}{2\zeta_{w}\sqrt{{\left(1-\frac{\omega_{w}^{2}}{\omega_{H}^{2}}\right)}^{2}+{\left(2\zeta_{U}\frac{\omega_{w}}{\omega_{H}}\right)}^{2}}}$$

while a perfectly specular expression can be obtained when $\omega =\omega_{H}$

(28) $${\left.\frac{U_{max}}{U_{\mathrm{inc}}}\right|}_{\omega =\omega_{H}}^{CF=0}=\frac{1}{2\zeta_{U}\sqrt{{\left(1-\frac{\omega_{H}^{2}}{\omega_{w}^{2}}\right)}^{2}+{\left(2\zeta_{w}\frac{\omega_{H}}{\omega_{w}}\right)}^{2}}}$$

**Figure 6:**
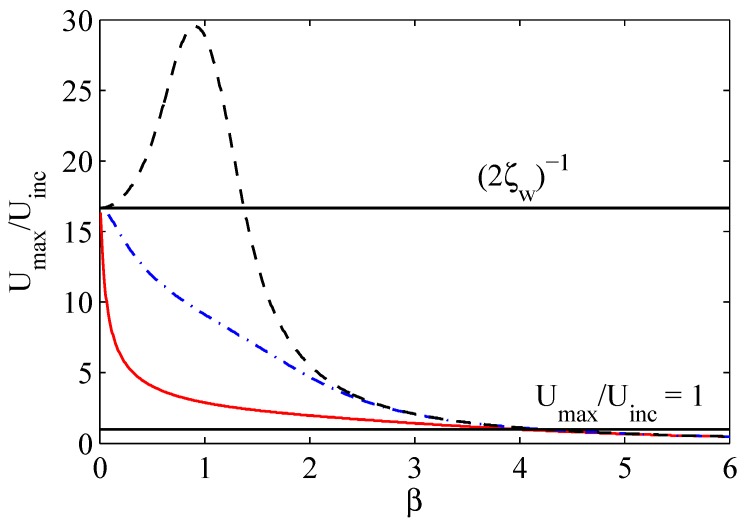
Amplification factor of jet peak velocity for decoupled oscillators (CF=0) and $\omega =\omega_{w}$, Equation (29). Continuous red line is for $\zeta_{U_{\mathrm{inc}}}=1$, dotted-dashed blue line for $\zeta_{U_{\mathrm{inc}}}=0.1$, dashed black line for $\zeta_{U_{\mathrm{inc}}}=0.01$.

Note that Equations (27) and (28) resemble very closely the canonical response of a damped spring-mass oscillator and that the ratio $\omega_{H}/\omega_{w}$ can be related to the CF parameter introduced before. Equation (27) can be rearranged to give a compact closed-form expression for the amplification factor

(29) $${\left.\frac{U_{max}^{2}}{U_{\mathrm{inc}}^{2}}\right|}_{\omega =\omega_{w}}^{CF=0}=\frac{-\zeta_{w}^{2}{\left(1-\beta^{2}\right)}^{2}+\sqrt{\zeta_{w}^{4}{\left(1-\beta^{2}\right)}^{4}+4\zeta_{w}^{2}\zeta_{U_{\mathrm{inc}}}^{2}\beta^{2}}}{8\zeta_{w}^{2}\zeta_{U_{\mathrm{inc}}}^{2}\beta^{2}}$$

where $\beta =\frac{\omega_{w}}{\omega_{H}}$ and $\zeta_{U_{\mathrm{inc}}}=\frac{KU_{\mathrm{inc}}}{2\omega_{H}l_{e}}$. The amplification factor given in Equation (29) depends exclusively on three non-dimensional parameters: β, $\zeta_{w}$, and $\zeta_{U_{\mathrm{inc}}}$. Since the structural damping factor is typically equal to 0.03, the acoustic damping and the resonance frequencies ratio can be used as free design parameters to optimize the actuator response. In Figure 6, Equation (29) is plotted as a function of β for three values of $\zeta_{U_{\mathrm{inc}}}$. Apparently, for very small coupling factors and in the (common) case of $\omega =\omega_{w}$, it is convenient to design the actuator so to have a small resonance frequencies ratio and (clearly) as small acoustic damping factors as possible. Also, Figure 6 shows that for the cases here analyzed, an actuator having β higher than about 4 is inconvenient since no amplification occurs. However, the above reasoning has to be augmented with efficiency considerations that will be made in Section 5.

Phase angles (with respect to the input supply) can also be computed. The diaphragm velocity phase angle $\phi_{V_{w}}$ reads:

(30) $$\phi_{V_{w}}=\tan^{-1}\left[\frac{2\zeta_{w}\frac{\omega }{\omega_{w}}\delta_{H}^{2}+2\zeta_{U}\frac{\omega }{\omega_{H}}\delta_{H}\delta_{CF}+2\zeta_{U}\frac{\omega }{\omega_{H}}\delta_{1}\delta_{2}+8\zeta_{w}\zeta_{U}^{2}\frac{\omega }{\omega_{w}}\frac{\omega^{2}}{\omega_{H}^{2}}}{\delta_{1}\delta_{2}\delta_{H}-4\zeta_{U}^{2}\frac{\omega^{2}}{\omega_{H}^{2}}\delta_{CF}}\right]$$

Additional insights can be obtained by again considering CF=0,

(31) $${\left.\phi_{V_{w}}\right|}^{CF=0}=\tan^{-1}\left[\frac{2\zeta_{w}\frac{\omega }{\omega_{w}}\delta_{H}^{2}+4\zeta_{U}\frac{\omega }{\omega_{H}}\delta_{H}\delta_{w}+8\zeta_{w}\zeta_{U}^{2}\frac{\omega }{\omega_{w}}\frac{\omega^{2}}{\omega_{H}^{2}}}{\delta_{w}\delta_{H}^{2}-4\zeta_{U}^{2}\frac{\omega^{2}}{\omega_{H}^{2}}\delta_{w}}\right]$$

It is easy to check from Equation (31) that when the diaphragm is driven at the structural resonance frequency, i.e., $\omega =\omega_{w}$, the phase angle of the diaphragm equals π/2,

(32) $${\left.\phi_{V_{w}}\right|}_{\omega =\omega_{w}}^{CF=0}=\frac{\pi }{2}$$

For what concerns the phase angle of the jet velocity, its general expression reads

(33) $$\phi_{U}=\tan^{-1}\left[\frac{2\zeta_{w}\frac{\omega }{\omega_{w}}\delta_{H}+2\zeta_{U}\frac{\omega }{\omega_{H}}\delta_{CF}}{\delta_{1}\delta_{2}+4\zeta_{w}\zeta_{U}\frac{\omega }{\omega_{w}}\frac{\omega }{\omega_{H}}}\right]$$

Again, under the hypothesis of decoupled oscillators, one has:

(34) $${\left.\phi_{U}\right|}^{CF=0}=\tan^{-1}\left[\frac{2\zeta_{w}\frac{\omega }{\omega_{w}}\delta_{H}+2\zeta_{U}\frac{\omega }{\omega_{H}}\delta_{w}}{\delta_{w}\delta_{H}+4\zeta_{w}\zeta_{U}\frac{\omega }{\omega_{w}}\frac{\omega }{\omega_{H}}}\right]$$

The phase angle occurring when the system is driven at $\omega =\omega_{w}$ reads:

(35) $${\left.\phi_{U}\right|}_{\omega =\omega_{w}}^{CF=0}=\tan^{-1}\left[\frac{1-\beta^{2}}{2\zeta_{U}\beta }\right]$$

Upon inspection of Equations (31)–(35), several considerations can be drawn for the common case in which the system is driven at the structural resonance frequency:

- if the structural resonance frequency equals the Helmholtz frequency (i.e., β=1), there is a π/2 phase difference between the diaphragm and the jet,
  (36) $${\left[\phi_{V_{w}}-\phi_{U}\right]}_{\omega =\omega_{w}}^{CF=0}=\frac{\pi }{2}\;\;\;\beta =1$$
- if the structural frequency is much higher than the resonance frequency (β≫1), then:
  (37) $$\lim_{\beta \to +\infty }{\left.\phi_{U}\right|}_{\omega =\omega_{w}}^{CF=0}=-\frac{\pi }{2}$$
  and the diaphragm and the jet are out of phase by π
  (38) $${\left[\phi_{V_{w}}-\phi_{U}\right]}_{\omega =\omega_{w}}^{CF=0}=\pi \;\;\;\beta \gg 1$$
- if the structural resonance frequency is very small (β≪1), then the compressible effects are negligible and the diaphragm and the jet are in phase with each other:
  (39) $${\left[\phi_{V_{w}}-\phi_{U}\right]}_{\omega =\omega_{w}}^{CF=0}=0\;\;\;\beta \ll 1$$

**Figure 7:**
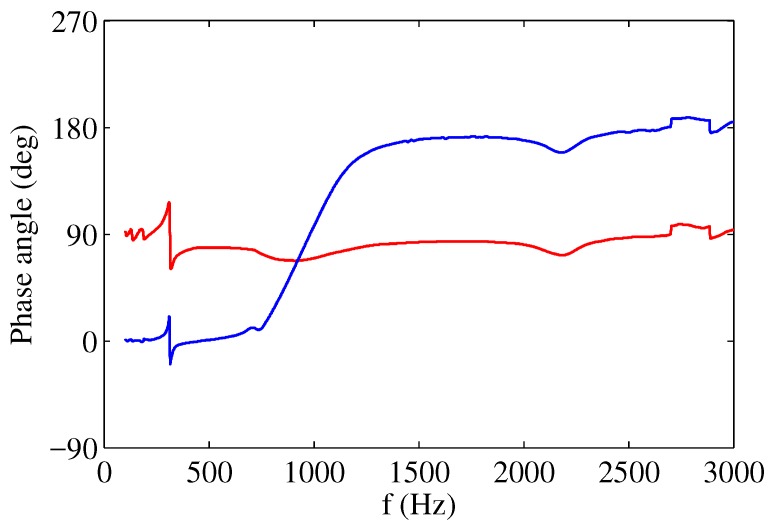
Computed phase angles for case 1 device of Gallas et al. [23]. Blue line refers to jet velocity—diaphragm velocity responses; red line to jet velocity - cavity pressure responses. Reprinted with permission from [31].

Similar considerations can be drawn for the general expressions given in Equations (30)–(33), for the limiting cases in which the system is driven at very low or very high frequencies, i.e., $\omega /\omega_{H}\ll 1$ or $\omega /\omega_{H}\gg 1$. The above results are in close qualitative agreement with the results obtained by Sharma [31] and reported in Figure 7.

### 2.3. Transduction Approach

A useful tool to study the behavior of an actuator, once it has been represented into a lumped mechanical system (basically including the oscillating diaphragm, the cavity and the orifice), consists in using the electric-mechanical analogy. This method considers the construction of an equivalent circuit in which an inductor represents the lumped mass (essentially the diaphragm), a capacitor reproduces the effects of the compliance (representing the elasticity of both the diaphragm and air), and a resistor symbolizes the damper (representing the structural and fluidic dissipation causes). In LEM formulation, these basic elements are able to exchange energy among themselves. In general, different energy domains can be introduced, and for each one, an effort e(t) and a flow f(t) are conveniently defined, see Mohseni and Mittal [17] and Senturia [38]. The power flow from one element to the other is given by the product of the effort by the corresponding flow. A useful correspondence for SJ devices is listed in Table 1.

A synthetic jet actuator is an electroacoustic device which involves different forms of energy, such as, electrical (input power), electrodynamical (stored in the diaphragm) and kinetic (of the air jet), Girfoglio et al. [39]. Following the work of Gallas [23], the changes between these different forms can be analyzed by using a two-port network where elements sharing a common effort (i.e., pressure or voltage) are connected in parallel, whereas those sharing a common flow (i.e., air volumetric flow rate or current) are connected in series. Therefore, the problem concerns the representation of these elements in the acoustic/fluidic domain and the determination of the corresponding electric impedances in the electric one. In LEM formulation, a device can be represented by two-coupled lumped mechanical systems (both of mass-spring-damper type), namely the diaphragm and the cavity/orifice, in which the kinetic energy is associated to a lumped mass *m*, the potential energy to a lumped spring of stiffness *k* (or compliance, $C=A^{2}/k$, according to Equation (9)) and the dissipation to a lumped dumper of coefficient *c*, as sketched in Figure 5. For the sake of clarity the reader is referred also to Equation (22).

**Figure 8:**
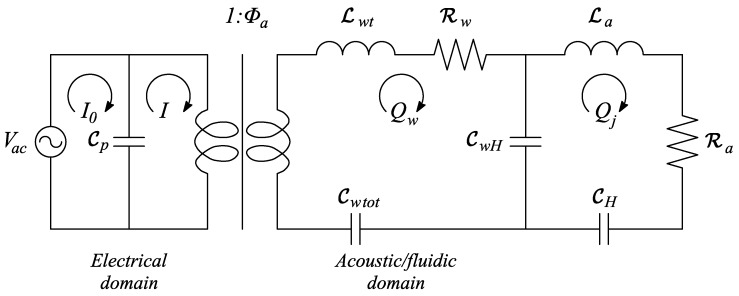
Electro-mechanical analogy.

In case the SJ is realized with an electromechanical element, such as a piezoelectric thin disk, the equivalent electric circuit is depicted in Figure 8, which corresponds strictly to the mechanical system of Figure 5, where the transduction from the electrical to the acoustic/fluidic domain is represented through an ideal transformer, having the transformation ratio equal to the electroacoustic transduction coefficient, $\Phi_{a}$. Within the framework of the mass-inductance analogy, the inductances $L_{w}$ and $L_{a}$ correspond to the diaphragm and orifice air masses, respectively, the capacitances $C_{p}$, $C_{wtot}$, $C_{wH}$ and $C_{H}$ correspond to the blocked electrical capacitance of the piezoelectric diaphragm (for more details see Prasad et al. [22]), the total diaphragm stiffness (including both parallel structural and acoustic stiffnesses, $k_{w}$ and $k_{w}^{\prime }$, respectively), the coupling stiffness and the Helmholtz stiffness, respectively, and the resistances $R_{w}$ and $R_{a}$ correspond to the structural and fluidic damping factors, respectively.

In fact, for an ideal transformer with unit electrodynamic transduction efficiency, i.e., the Joule power provided to the piezo-element equals the electrodynamic power (detailed insights on the actuators energy efficiency will be discussed in Section 5), it holds:

(40) $$V_{ac}I=F{\dot{x}}_{w}$$

which can be also written in terms of cavity pressure and wall volumetric flow rate $Q_{w}={\dot{x}}_{w}A_{w}$

(41) $$V_{ac}I=p_{i}Q_{w}$$

By taking into account that the driving force magnitude is expressed by Equation (4), and by considering the relationships given in Equations (7) and (9), one obtains finally

(42) $$p_{i}/V_{ac}=\Phi_{a}$$

As already stated above, the solution to the electric LEM equations is usually expressed in terms of periodic stationary behavior of the actuator, where the ratio of the output air jet flow rate to the input voltage is given by the equivalent impedance. With reference to Figure 8, and following Gallas et al. [23], denoted with $Z_{w}$, $Z_{c}$, and $Z_{o}$ the total impedances of the diaphragm, cavity, and orifice respectively, one obtains:

(43) $$\frac{Q_{j}}{V_{ac}}=\frac{Z_{c}}{Z_{w}Z_{o}+Z_{w}Z_{c}+Z_{c}Z_{o}}$$

where $Q_{j}$ is the volumetric flow rate of air issuing from the orifice (i.e., corresponding to the air jet velocity *U*). A major goal of the device design is to maximize the magnitude of the volumetric flow rate through the orifice per applied voltage, $|Q_{j}/V_{ac}|$. It has to be stressed that for the approach followed in the present paper the relevant impedances are directly related to the mechanical quantities defined, for instance, in Figure 5; however, within the equivalent circuit model analysis, such impedances are evaluated in a specific way component by component, as made by Gallas et al. [23] and Mohseni and Mittal [17]. The way in which the electric circuit LEMs evaluate the impedances makes these approaches different from the fluid dynamic-based LEMs.

Starting from Equation (43) it is possible in principle to calculate the coupled resonance frequencies of the system. This analysis has been performed by Gallas et al. [23], who developed a non-closed form procedure to obtain such frequencies, under the assumption of small damping effects, and compared the computed frequencies with experimental data, as well as with the corresponding uncoupled values. The approach of Gallas et al. [23] is based on the already mentioned relationship $\omega_{1}\omega_{2}=\omega_{w}\omega_{H}$, where $\omega_{1}$ and $\omega_{2}$ are the coupled natural frequencies. The reader is referred to the original paper to obtain more details. Note that in the present paper the relationships of de Luca et al. [32] have been already reported in Equation (15) which, in agreement with the constraint of Gallas et al. [23], predicts that the coupling effect augments the greatest uncoupled frequency and reduces the lowest one. Additional information about the evaluation of the two modified resonance frequencies can be found in Persoons [25], who employed a simplified heuristic argument and referred to loudspeaker-driven actuators.

### 2.4. Dimensionless Equations

With the aim of providing further insights to the problem physics, the governing equations analyzed previously can be recast into a convenient dimensionless form, de Luca et al. [33].

The nondimensionalization process relies on the choice of proper reference scales for time, length and velocity; for the equation of dynamics of the acoustic oscillator, Equation (11), a convenient set of quantities is represented by the reciprocal of the operating frequency 1/ω, the cavity height *H* and the air speed of sound *c*, respectively. The nondimensional form of the equation is thus

(44) $$St^{2}\left(\frac{l_{e}}{H}\right)\frac{d^{2}U^{*}}{dt^{*2}}+St\left(K|U^{*}|\right)\frac{dU^{*}}{dt^{*}}=V_{w}^{*}-\frac{A_{o}}{A_{w}}U^{*}$$

where the Strouhal number, defined as:

(45) $$St=\frac{\omega H}{c}$$

can be re-written in function of other parameters:

(46) $$St=\frac{\omega }{\omega_{H}}\frac{d_{o}}{d_{w}}\sqrt{\frac{H}{l_{e}}}$$

The condition St≪1 represents the case of acoustically thin cavity, for which the traveling time of a small pressure disturbance, over the distance *H*, is much smaller than the reference time 1/ω. From an operative point of view, the air inside the cavity behaves as an incompressible medium (i.e., the air stiffness is infinite), and Equation (44) in this case reduces to the (dimensional) relationship:

(47) $$A_{w}V_{w}=A_{o}U$$

This means that the volume flow rate entering the cavity, as a consequence of the diaphragm displacement, equals the volume flow rate of air expelled through the orifice. On the other hand, Equation (1) shows that the diaphragm dynamics is decoupled from that of the acoustic oscillator, with the diaphragm being driven by the piezoelectric forcing only. One can easily verify this result by examining the electric circuit of Figure 8, for a null air capacitance (or null air elastic compliance). When St≪1, once the air velocity at the orifice has been obtained from Equation (47), the cavity pressure may be evaluated by using the unsteady form of the Bernoulli’s equation. In the following, one will refer to Equation (47) as the *incompressible model* of operation.

It is also convenient to observe that, for small concentrated head losses, the driving differential pressure $p_{i}$ has to be of the same order as the dynamic pressure at the orifice in the Bernoulli’s equation, that allows the determination of a more appropriate scale velocity of the orifice jet, $U_{ref}$. Since, through the isentropic transformation of perfect gas, the pressure variation related to the orifice volume variation $A_{o}dx$, with dx being the axial variation scaling with $l_{e}$, scales with $\gamma \frac{p_{a}}{V_{c}}A_{o}l_{e}$, one finally obtains $U_{ref}=c\frac{d_{o}}{d_{w}}\sqrt{\frac{l_{e}}{H}}$. As a consequence, it is easy to verify that the Strouhal number is directly represented by the ratio $St=\frac{\omega }{\omega_{H}}$, namely formally by $St=\frac{\omega l_{e}}{U_{ref}}$.

Another relevant condition occurs for St≫1, which also corresponds to decoupled diaphragm dynamics. In this case the air stiffness is vanishing, the pressure field inside the cavity is practically unperturbed, and therefore the air jet velocity U is vanishing too. Again one can uncover this situation by taking a look at Figure 8. It is worth noting that for this condition the lumped assumption is of course invalid, hence it has not been considered during the device performance evaluation (Section 4). However, the Strouhal number is quite less than unity even at the highest operation frequencies generally considered in the plots shown in the paper; for instance, for H=1.5mm and ω=2500Hz it results $St≃6.93\times 10^{-2}$.

Equation (13) is made dimensionless with the introduction of different time and velocity scales; here, convenient choices are $1/\omega_{w}$ and $\omega \Delta x_{w}$, respectively. The non-dimensional form of such an equation is:

(48) $${\ddot{V}}_{w}^{*}+2\zeta_{w}{\dot{V}}_{w}^{*}+V_{w}^{*}+CF\left(V_{w}^{*}-\frac{A_{o}}{A_{w}}U^{*}\right)=\cos\omega t$$

where the coupling parameter CF has been introduced in Equation (25). Note that under the condition CF≪1 (which means that the air stiffness is negligible in comparison with the diaphragm stiffness) the diaphragm dynamics is decoupled from the acoustic oscillator one. In this case the jet velocity and the cavity pressure are determined via the continuity and the unsteady Bernoulli’s equations. Furthermore, the modified structural and Helmholtz’s frequencies tend to coincide with the corresponding uncoupled frequencies, as one may verify by inspecting Equation (15). It has to be also stressed that, in general, the coupling effects represented by the CF parameter refer to a certain device and may be neglected on the basis of design characteristics of the actuator, whatever is the operating condition. On the contrary, the conditions of decoupling occurring for St≪1 depend basically on the operating condition and may occur for any device.

The inclusion of the effects of the external viscous medium (air) leads to the introduction of the jet Reynolds and Stokes numbers (e.g., Smith and Glezer [3])), $Re=\frac{\bar{U}d_{o}}{\nu }$ and $S=\frac{\omega d_{o}^{2}}{\nu }$, as indicated in Table 2, which summarizes all the nondimensional parameters affecting the actuator operation (with $d_{o}$ and $d_{w}$ being the orifice diameter and the diaphragm diameter, respectively).

## 3. Comparison of LEM Results

**Figure 9:**
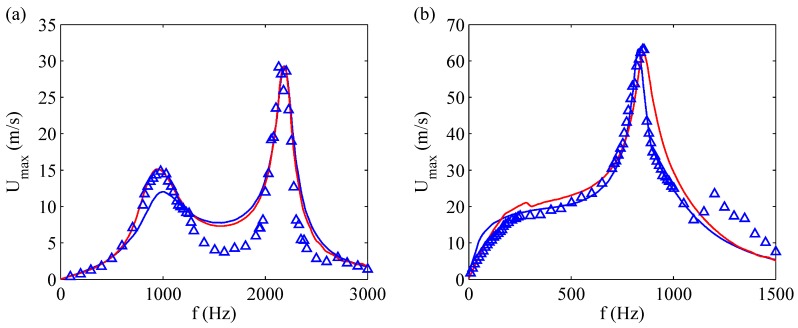
Comparison of experimental data and Lumped Element Modeling (LEM) results of peak jet velocity for two actuators, (a) case 1 and (b) case 2, reprinted from [31]. Blue markers are experimental data from Gallas et al. [23], blue line represents LEM by Gallas et al. [23], red line LEM by Sharma [31]. Reprinted with permission from [31].

Very interesting comparisons of LEM results obtained by means of both the mechanical and electric approach were showed by Sharma [31], who computed the frequency response of the devices tested by Gallas et al. [23], and compared his results to the stationary solutions of Gallas et al. [23], obtained applying directly the method of the electrical impedances. Therefore, the comparison is meaningful because the models of Sharma [31] and Gallas et al. [23] are quite different, but they were applied to the very same physical system. Figure 9 depicts the centerline maximum jet velocity (achieved during the periodic stationary operation phase) as a function of the operating frequency of two different actuators, named case 1 and case 2 of Gallas et al. [23], who made use of a Dantec two-component laser Doppler velocimetry system to measure the peak centerline jet velocity $U_{\max}$. These authors took care of measuring the jet velocity at an axial station very close to the orifice, or in other terms, such that the ratio of the measurement location to the stroke length was much less than unity. As a consequence, they could compare directly the measured peak velocity to $U_{\max}$ provided by LEM.

Note that both models reproduce well the experimental data concerned with the two resonance frequencies and the corresponding velocity peaks, although in the case of device 1 the numerical findings overestimate the experimental data points in between the two peak frequencies. For the case of device 2 two resonance frequencies are predicted by the electrical LEM, one near 350 Hz and the other one near 900 Hz. However, the lower peak is heavily damped due to the nonlinear fluidic damping acting on the acoustic oscillator. The major geometrical and electro-mechanical characteristics of the devices designed by Gallas et al. [23] are shown in Table 3.

**Figure 10:**
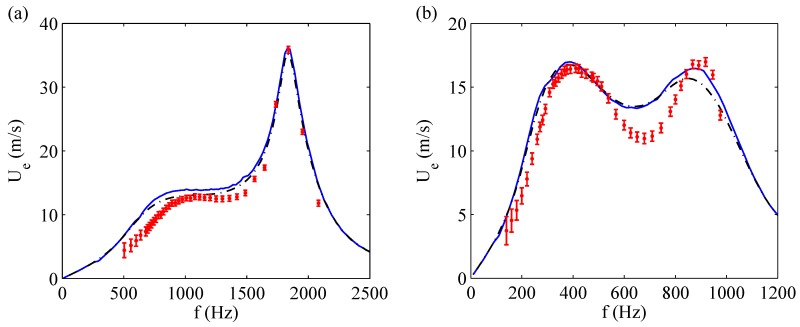
Numerical, analytical and experimental comparison of saddle point velocity for (**a**) the brass actuator (H/do=0.75, $V_{ac}=50$ V) and (**b**) the aluminum actuator (H/do=0.8, $V_{ac}=50$ V). Blue solid lines are numerical results, black dash-dotted curves analytical ones and red markers experimental data with their uncertainty bars. Reprinted with permission from [33].

Figure 10 reports other comparisons made by de Luca et al. [33] for devices with the shim in brass, frame (a), and aluminum, frame (b), respectively. Each picture compares the experimental data with the findings obtained by means of the fully fluid dynamics modeling (where the stationary solution was obtained by integrating the governing equations for long times), as well as with the *modal* stationary solution, that is an equivalent form of the electric LEM approach. In this case, however, the impedances are obtained from the fluid dynamics approach.

**Figure 11:**
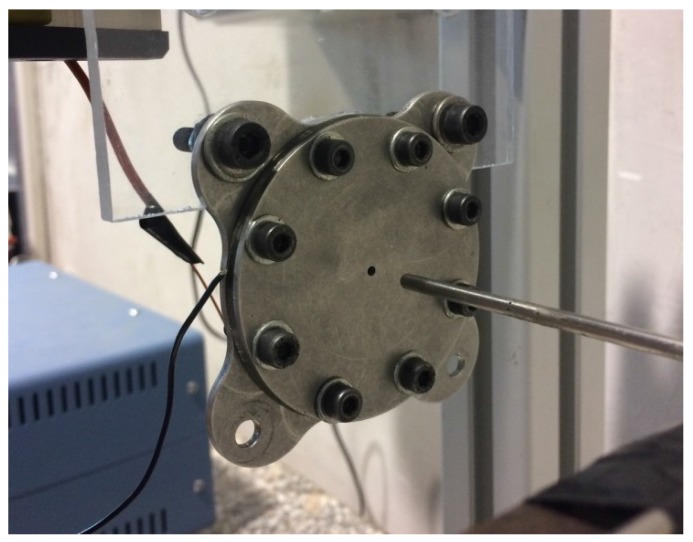
Experimental mock-up with Pitot tube.

The experimental mock-up is reported in Figure 11 showing in particular the Pitot tube (Kanomax mini Pitot tube, 1/8in in diameter and 6in in length) employed to measure the jet velocity. The actuator has been electrically excited with a sine signal generated through a USB Instruments DS1M12 or “Stingray” (USB Instruments – EasySync Ltd., Glasgow, UK) (which can work simultaneously as both signal generator and data-acquisition system) and then transmitted to a linear gain amplifier (Piezo Linear Amplifier EPA-104, Piezo Systems, Inc., Woburn, MA, USA). Exit jet velocities were measured in the external ambient at the saddle point, therefore in this study velocity experimental measurements $U_{e}$ and LEM predictions of peak jet velocity $U_{\max}$ were correlated to each other by means of the relationship of Equation (21). The Pitot tube was connected with a Mouser pressure transducer (“HSCDRRN002NDAA5” and “HSCDRRN005NDAA5”) whose output signal was phase-locked to the device excitation signal over typically 100 periods and the uncertainty has been estimated with standard procedures, see Moffat [40]. The agreement between numerical and analytical solutions is fully satisfactory for the brass shim, while for the aluminum shim actuator one can note again an over-prediction of the theoretical results with respect to the experimental data at intermediate frequencies. The geometrical and electro-mechanical characteristics of the devices designed for this purpose are shown in Table 4.

## 4. Performances

The various physical models have been validated against experimental measurements and the relevant comparisons have been shown in the previous sections. LEM will be used hereafter to predict the influence of the coupling degree of the two oscillators (via the Strouhal number and the coupling factor CF), and of other parameters such as the supply voltage and the effective orifice length.

### 4.1. Effect of the Coupling of the Oscillators

The effect of the Strouhal number can be appreciated by focusing the attention on particular operating conditions; for a given device the condition of St≪1, that is achieved for relatively low values of operation frequency, corresponds to situations of acoustically thin resonant cavity, as observed before. This effect will be analyzed numerically with particular reference to the frequency response in terms of exit velocity of the three devices mentioned in the previous sections.

**Figure 12:**
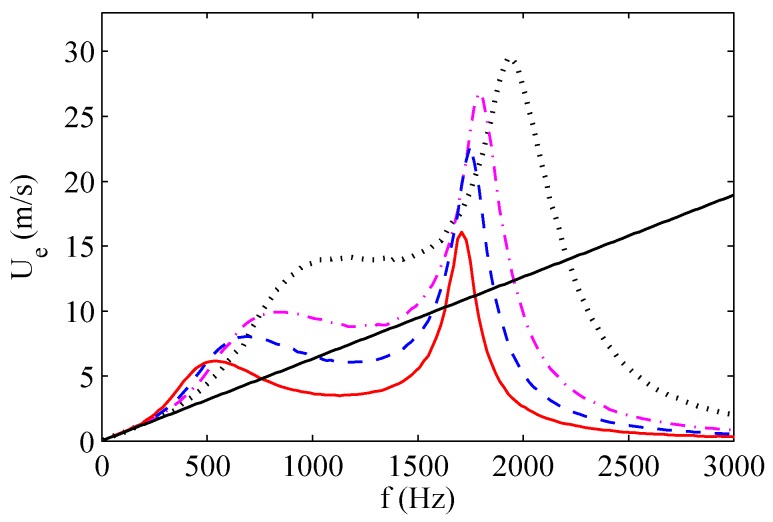
Frequency response of saddle point velocity for the brass actuator at $V_{a}=35$ V; black dotted line refers to $H/d_{o}=0.5$, magenta dash-dotted line to $H/d_{o}=1$, blue dashed line to $H/d_{o}=\;1.5$, red solid line to $H/d_{o}=2.5$. The straight line refers to Equation (47). Reprinted with permission from [33].

**Figure 13:**
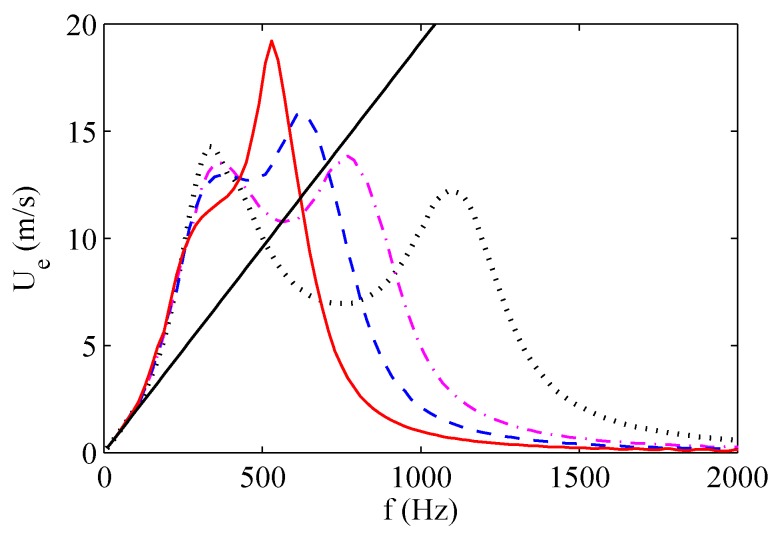
Frequency response of saddle point velocity for the aluminum actuator at $V_{a}=35$ V; black dotted line refers to $H/d_{o}=0.5$, magenta dash-dotted line to $H/d_{o}=1$, blue dashed line to $H/d_{o}=1.5$, red solid line to $H/d_{o}=2.5$. The straight line refers to Equation (47). Reprinted with permission from [33].

The maximum exit velocity trends as functions of the operation frequency are depicted in Figure 12 and Figure 13 for two of the three devices tested by de Luca et al. [33], whose characteristics are listed in Table 4, and for various dimensionless cavity heights $H/d_{o}$. The supply voltage $V_{a}$ is equal to 35 V in all these simulations.

For all the devices, two velocity peaks corresponding to the two resonance frequencies are clearly evident. For the brass device, both velocity peaks increase with decreasing cavity height. The distance between the two resonance frequencies slightly increases with increasing $H/d_{0}$, in agreement with experimental results of Gomes et al. [37] obtained for $l_{o}/d_{o}>1$, reported in previous Figure 4. Note that the experimental findings of Gomes et al. [37] show also that the distance between the resonance frequencies becomes practically constant as $H/d_{0}$ further increases, in agreement with the analytical prediction of de Luca et al. [32] valid in the case of $\omega_{w}\gg \omega_{H}$.

The straight lines present in the plots of Figure 12 and Figure 13 refer to the linear dependence of the jet velocity upon the operating frequency given by the incompressible model described by the Equation (47). For both actuators it is clearly evident that such a simplified model closely agrees with the simulations of the complete model at low frequencies, with the frequencies range of such an agreement widening for the smaller cavity heights, as predicted by the theory for St≪1. Note also that for this range of frequencies the response in terms of jet velocity is the same whatever is the cavity height, thus confirming that the diaphragm dynamics is decoupled from the acoustic oscillator one.

**Figure 14:**
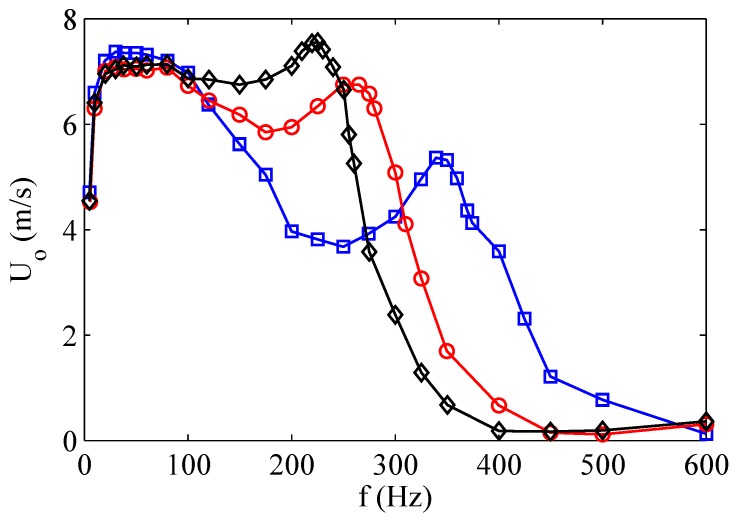
Spatially- and temporally-averaged *momentum velocity* for various cavity heights. Blue square markers are for H=20 mm, red circles for H=40 mm and black diamonds for H=60 mm. Reprinted with permission from [41].

In order to complete the discussion about the behavior of the aluminum device, note that the two nominal Helmholtz and structural frequencies, which for this actuator are reversed, are remarkably modified by the high coupling ratio. The jet velocity decreases with increasing the cavity height at the structural resonance frequency, whereas it increases with increasing $H/d_{o}$ at the Helmholtz resonance frequency, with the result that the maximum peak is reached at the Helmholtz frequency for the highest simulated cavity height. This particular finding agrees with the theoretical prediction of de Luca et al. [32] that if $\omega_{w}\ll \omega_{H}$ then the distance between the two eigenvalues $|\omega_{1}^{2}-\omega_{2}^{2}|/\omega_{H}^{2}$ does not depend on the cavity height $H/d_{o}$ and therefore $|\omega_{1}^{2}-\omega_{2}^{2}|≃1/\left(H/d_{o}\right)$. The quasi-coincidence of the two resonance frequencies justifies that the maximum peak is reached for the highest cavity height. As already observed, this last result has been confirmed experimentally by Gil and Strzelczyk [41] for the case of loudspeaker driven actuators, as depicted in Figure 14. Note that these authors referred to a spatially- and temporally-averaged *momentum velocity* $U_{o}$.

In Section 2.2 the magnification of the device response with respect to the so called incompressible solution has been analyzed theoretically, in connection with the role played by the simultaneous presence of the two damping effects, with particular emphasis to that of the nonlinear fluidic term.

By resorting to the classic linear theory of damped-driven harmonic oscillators, under the simplified assumption of decoupled damping effects, de Luca et al. [33] defined the acoustic oscillator as *under-damped* or *over-damped* depending on whether the Acoustics Damping Coefficient (ADC), ADC$\;=\;K\left(|\tilde{U}|/l_{e}\right)/\left(2\omega_{h}\right)$, is less than or greater than unity, respectively. Note that here ADC corresponds to the fluidic damping factor $\zeta_{U}=\left(U_{\max}K\right)/\left(2\omega_{H}l_{e}\right)$ previously introducted in Section 2.2. The under-damped case corresponds to the occurrence of resonance amplifications and therefore, if one extends such implications to the present nonlinear case, the jet should clearly form when driven at the Helmholtz frequency. Due to the nonlinearity in jet velocity *U*, de Luca et al. [33] argued that the jet velocity should refer to a proper scaling value, namely to the average value, $\tilde{U}$, giving the equivalent amount of energy dissipated in a quarter of period by the variable damping coefficient. It is easy to verify that $\tilde{U}=U_{\max}/2$, with $U_{\max}$ denoting the maximum value reached in the cycle. These predictions were qualitatively confirmed by the numerical simulations referring as usual to brass and aluminum actuators. For each device the diaphragm peak velocity, the average jet velocity, and ADC, were shown, as functions of the operating frequency, for various values of the head loss coefficient (mostly non-physical).

Here it should be stressed the crucial role played by the nonlinear flow damping on the amplitude of the oscillations, as is well recognized in the literature, e.g., by Gallas et al. [23] (who attributed to it the absence of the first resonance peak for their device of case 2, see the right frame of Figure 9), Persoons [25], Kooijman and Ouweltjes [42]. de Luca et al. [33] depicted also diaphragm velocity and jet velocity, which were evaluated by means of the incompressible model of Equation (47). Remember that the incompressible model furnishes the reference value to be used to evaluate the amplification of the oscillations amplitude due to the resonance, as discussed in depth in Section 2.2, where both the structural and fluidic damping effects are simultaneously taken into account, within a generalized nondimensional context.

As far as the brass device is concerned, de Luca et al. [33] confirmed that, due to the vanishing coupling factor, the diaphragm dynamics is decoupled from the acoustic oscillator. In fact, the trends of the diaphragm velocity are shown to be practically independent of the head loss coefficient, there is no resonance at the Helmholtz frequency (about 1000 Hz) and the incompressible model applies well up to frequencies not too greater than the Helmholtz frequency. Moreover, the acoustic oscillator is always forced by the diaphragm one, and exhibits two resonance peaks, at almost decoupled Helmholtz and structural frequencies. For the aluminum device the oscillators are fully coupled, as one expects from the relatively high coupling factor CF=1.38. In fact, the diaphragm velocity exhibits two resonance peaks (remember that for this device the structural resonance frequency is less than the Helmholtz one) and both velocity resonance peaks are influenced by the nonlinear damping coefficient.

### 4.2. Effect of the Voltage

**Figure 15:**
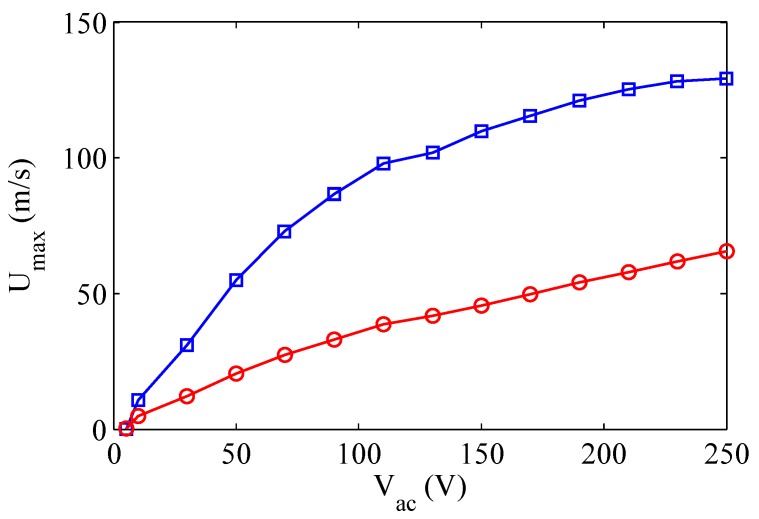
SJ peak velocity response as a function of excitation frequency at resonance frequencies ($h/d_{o}=2.1$ and $H/d_{o}=0.56$). Blue data points are for diaphragm resonance, red data for acoustic resonance. Reprinted with permission from [43].

Based on the scaling relationships of the jet velocity shown in Section 2.2, for instance with reference to Equation (29), remembering that $U_{inc}$ scales with $\Delta x_{w}$ (which in turn is proportional to $V_{ac}$ through Equation (4)), it is possible to argue that the jet velocity basically depends linearly on the supply voltage, apart from a weak nonlinear influence due to the fluidic damping term. Moreover, due again to the damping effects, as the voltage increases a corresponding reduction of the distance between the (coupled) resonance frequencies is observed in LEM simulations (not shown herein). This agrees with the experimental measurements reported by Crowther and Gomes [43], who studied the frequency response of SJ actuators by varying the supply voltage. However, it has to be pointed out that for relatively high voltage values the benefit of higher velocities is remarkably reduced. It is believed that this behavior is due to the gradual saturation of the PZT material (as addressed also by Krishnan and Mohseni [36]). Hence, in practice the velocity amplitude does not exhibit a linear behavior with increasing the excitation amplitude, as depicted in Figure 15 reprinted from Crowther and Gomes [43]. On the other hand, these authors asserted also that measurements of the diaphragm peak displacement at a high excitation voltage ($V_{ac}=250$ V) show that this is of the order of 75μm, which is well within the expected linear stiffness region of the diaphragm, so this is unlikely to contribute towards saturation. Therefore, they concluded that “there would be non-linear fluid dynamic losses due to compressibility and viscous effects as the flow discharges through the orifice”.

**Figure 16:**
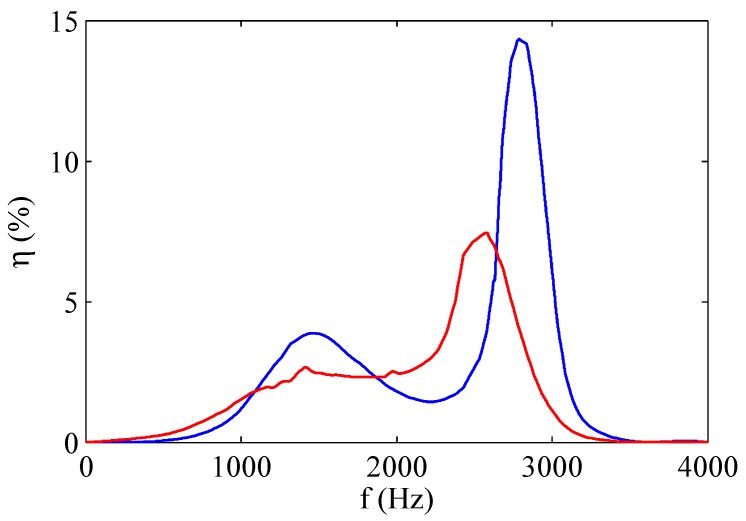
Electric–fluid energy conversion efficiency as a function of excitation frequency. Blue line refers to $V_{ac}=90$ V, red line to $V_{ac}=250$ V ($h/d_{0}=2.1$ and $H/d_{0}=0.56$). Reprinted with permission from [43].

Crowther and Gomes [43] faced also the influence of the electric voltage on the energy efficiency, i.e., the ratio of the air kinetic energy issuing from the orifice to the input electric power, of SJ devices (which will be the topic of the next Section). They found experimentally that as the peak-to-peak excitation amplitude increases above 120 V, the energy efficiency η remarkably reduces, decreasing to a value of 7 per cent at the peak velocity output condition, i.e., $V_{ac}=250$ V, as shown in Figure 16.

Also Gil and Strzelczyk [41] described the effects of the supply voltage on the efficiency of the actuators, but their analysis refers to the case of loudspeaker-driven actuators and is restricted to very low values of voltage.

### 4.3. Effect of the Orifice Length

The effective orifice length, $l_{e}$, has a relatively strong effect, because it influences the magnitude of the jet velocity issuing from the orifice through the amount of the fluidic damping coefficient, and the nominal Helmholtz’s frequency, which is inversely proportional to the effective mass of the air at the orifice, $m_{a}=\rho_{a}l_{e}A_{o}$. The diaphragm structural frequency should be in principle independent of this parameter and therefore it should keep constant, apart from the influence of the coupled damping effects.

Gomes et al. [37] carried out an extensive experimental investigation about the influence of the orifice nominal depth $l_{o}$. They preliminarily claimed that there are two effects acting on the flow traveling through the orifice which are of importance to understand how the velocity behaves with increasing the orifice length: firstly, the shorter the extension of the orifice, the bigger the effect of vena contracta on the volume flow rate will be. Secondly, the boundary layer displacement thickness grows with length, due to the action of shear of the slow fluid near the wall on the faster flow in the core. This tends to slow down the core flow and hence to reduce the peak velocity. Therefore, there must be a compromise between these two phenomena such that an optimum can be reached. Gomes et al. [37] argued that the optimal length of the orifice would be the summation of the length required to achieve a flow marginally attached to exit edges of the orifice, so as to minimize the vena contracta effect (i.e., $l_{o}/d_{o}≃0.75$), plus the minimum length required for the flow to reattach after the bubble separation due to the vena contracta (about half exit diameter), resulting in a total orifice depth of around $l_{o}/d_{o}≃1.25$. The major findings of their experimental measurements are summarized as follows: the actuator peak velocity reduces with increasing the orifice depth; the unsteadiness of magnitude and position of the diaphragm resonance peak gradually reduces with increasing the orifice depth. The entire decoupling of cavity resonance from diaphragm resonance is gradually offset to lower frequencies with increasing $l_{o}/d_{o}$. This effect supports the theoretical prediction of the inverse square proportionality between the Helmholtz frequency value and the orifice depth.

**Figure 17:**
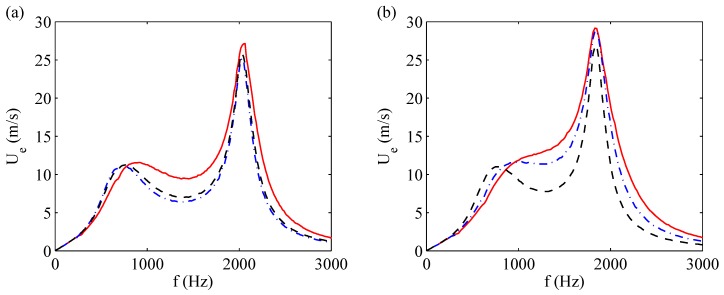
Frequency response of saddle point flow velocity for (**a**) the aluminum actuator and (**b**) the brass actuator at different equivalent length; red solid line refers to $l_{e}/d=1$, blue dashed line to $l_{e}/d=2$ and black dotted line to $l_{e}/d=3$.

Figure 17 report the frequency response of the exit flow velocity for two of the actuators tested by de Luca et al. [33] and frequently reconsidered in this paper, for various values of nondimensional effective length $l_{e}/d_{o}$. It is worth to remind the reader that the effective length of the orifice corresponds to the distance between the locations of application of the Bernoulli equation and influences the volume of air mass of the Helmholtz oscillator. In both cases here examined, the structural frequency remains almost unchanged, while the Helmholtz’s one attains lower values for higher effective orifice lengths; one should also remember that for the brass actuator the Helmholtz’s frequency corresponds to the first resonance peak, whilst for the aluminum device the situation is reversed. Furthermore, it is interesting also to observe that the corresponding jet velocity slightly diminishes at the Helmholtz frequencies for the brass actuator (in agreement with the findings of Gomes et al. [37]), but it increases for the case of aluminum. The peak velocity at structural resonance frequency decreases with increasing $l_{e}/d_{o}$ for aluminum actuator.

Gil and Strzelczyk [41] reported other experimental data on the effect of the orifice length, but their measurements refer to the case of loudspeaker-driven actuator.

## 5. Efficiency of Piezo-Driven Devices

Crowther and Gomes [43] studied the system costs associated with the application of flow control system to civil transport aircraft, based on the use of electrically powered synthetic jet actuators. They defined the efficiency of the actuator as the fluid power (scaling with the cube of the exit velocity) divided by absorbed electrical power, and analyzed it as a function of the operating conditions and actuator geometry, which were chosen reasonably close to those expected for industrial applications. For this reason, the experiments were basically carried out with a chamber depth to orifice diameter ratio equal to 0.56 and an orifice depth to diameter ratio equal to 2.1. Crowther and Gomes [43] considered that the difference between the supplied electrical power and the gained fluid power was lost because of electrical impedance (ascribed to the piezo-electric actuator), mechanical impedance (related to the diaphragm dynamics) and acoustic impedance (due to the fluid-acoustic coupling of the flow within the cavity and through the orifice), however without accurately quantifying each term of the balance. They showed their experimental findings in terms of a map of the electric-fluidic conversion efficiency, where such an efficiency was reported as a function of the excitation voltage amplitude (for peak-to-peak excitation voltage up to 250 V) and actuation frequency (up to 4000 Hz). They found that the efficiency attains a maximum equal to about 14% and noted that it did not correspond to the condition of maximum exit velocity because of the dielectric saturation effect (discussed in the previous section) affecting the commercial piezo-electric patch (i.e., the piezo-element bonded to the brass shim).

A more recent work was presented by Li et al. [44] who expressed on an analytical basis every term contributing to the energy rate balance. They argued that once the synthetic jet has received electric energy input, a part of the energy is stored as electric potential energy while the rest of the energy is converted to mechanical energy accompanied by energy dissipation. The mechanical energy includes vibration of piezo-electric actuators and kinetic energy of air flow. For piezo-electric actuators two forms of energy, i.e., strain energy and kinetic energy, are temporarily stored by the vibrating structure. Energy dissipation occurs in piezo-electric actuators due to the deflection dynamics, as well as in the air flow motion (i.e., the head losses) when traversing the jet orifice. The synthetic jet device efficiency is defined as the ratio of the kinetic power of the air flow to the input electric energy. The authors carried out experiments on two slot synthetic jets having orifice length of 4 mm and 15 mm, for two values of the voltage amplitude (80 V and 100 V) and actuation frequency ranging from 200 Hz to 1100 Hz. Li et al. [44] found that the efficiency of energy conversion is dependent on the orifice size and on the operating conditions (namely, voltage and frequency) showing a peak (of about 40% for the 15 mm orifice) close to the mechanical resonance frequency of the actuator.

In the attempt of assessing a rigorous and comprehensive physical approach to the evaluation of the efficiency of piezo-electrically driven synthetic jet actuators, based on a rather detailed modeling of the actuator dynamics, Girfoglio et al. [39] presented a novel approach where the energy balance equation, properly averaged over the actuation period, is derived directly from the equations governing the dynamics of the actuator. The (instantaneous) energy balance equation of the actuator system can be obtained by summing the equations of energy of the diaphragm (that takes into account both kinetic and elastic strain contributions) and of kinetic energy of the air mass through the orifice. Moreover, to characterize the actuator behavior over an operating cycle, it is convenient to apply the time average operator on the resulting equation, defined as:

(49) $$\bar{\varphi }=\frac{1}{T}\int_{0}^{T}\varphi dt$$

where φ is a generic time-dependent variable.

The energy balance equation averaged over an actuation cycle (i.e., over a time equal to the period *T*) is given by

(50) $$\begin{array}{c} \underset{\bar{\Delta E}}{\underset{︸}{\frac{1}{T}\int_{0}^{T}d\left(E_{w}+E_{o}\right)}}=\underset{{\bar{P}}_{e}}{\underset{︸}{\frac{1}{T}\int_{0}^{T}F{\dot{x}}_{w}dt}}+\underset{{\bar{P}}_{m}}{\underset{︸}{\frac{1}{T}\int_{0}^{T}p_{i}\left(A_{o}U-A_{w}{\dot{x}}_{w}\right)dt}}\\ \;\;\;\;\;\;\;\;\;\;\;\;-\underset{{\bar{D}}_{s}}{\underset{︸}{\frac{1}{T}\int_{0}^{T}c_{wt}{{\dot{x}}_{w}}^{2}dt}}-\underset{{\bar{D}}_{f}}{\underset{︸}{\frac{1}{T}\int_{0}^{T}\frac{1}{2}\left(K-1\right)\rho_{a}A_{o}{\left|U\right|}^{3}dt}}-\underset{{\bar{P}}_{k}}{\underset{︸}{\frac{1}{T}\int_{0}^{T}\frac{1}{2}\rho_{a}A_{o}{\left|U\right|}^{3}dt}} \end{array}$$

An accurate description of the various contributions of Equation (50) is reported hereafter:

- $\bar{\Delta E}$ is the total energy variation. Note that this term is null because, for each cycle, there is no change for $E_{w}$ and $E_{o}$, with $E_{w}$ and $E_{o}$ being the diaphragm energy and the kinetic energy of air issuing from the orifice respectively. The diaphragm energy takes into account both kinetic and elastic strain contributions.
- ${\bar{P}}_{e}$ is the electrodynamic power provided to the membrane by the applied voltage.
- ${\bar{P}}_{m}$ is the mechanical power due to the work done by the differential pressure $p_{i}$ which acts on the wall surface $A_{w}$ and on the orifice surface $A_{o}$. By using Equation (10), it can be shown that this term is proportional to $\frac{1}{2}\left({p_{i}}^{2}\left(T\right)-{p_{i}}^{2}\left(0\right)\right)$ and, therefore, it does not give any contribution because $p_{i}$ assumes the same value at the beginning and end of each cycle. One can reach the same result by observing that the pressure work is conservative by definition.
- ${\bar{D}}_{s}$ is the power dissipation due to the structural damping effects of the diaphragm. Note that, according to standard notations, $c_{wt}=2m_{wt}\zeta_{w}\omega_{w}$.
- ${\bar{D}}_{f}$ is the power dissipation due to the head loss of fluid dynamics type at the orifice.
- ${\bar{P}}_{k}$ is the kinetic power of air flow at the orifice. Note explicitly that the kinetic power here refers by definition to the entire cycle, i.e., suction phase included.

Then, by deleting $\bar{\Delta E}$ and ${\bar{P}}_{m}$ terms, Equation (50) becomes:

(51) $${\bar{P}}_{e}-{\bar{D}}_{s}-{\bar{D}}_{f}-{\bar{P}}_{k}=0$$

and, once defined the kinetic efficiency $\eta_{k}$ as the ratio of the kinetic power of the exit flow ${\bar{P}}_{k}$ to the electrodynamic power ${\bar{P}}_{e}$, one obtains:

(52) $$\eta_{k}=\frac{{\bar{P}}_{k}}{{\bar{P}}_{e}}=1-\frac{{\bar{D}}_{s}+{\bar{D}}_{f}}{{\bar{P}}_{e}}$$

In practice, the global efficiency of an actuator has to quantify the amount of Joule power provided to the system ${\bar{P}}_{j}$ that is actually converted in ${\bar{P}}_{e}$. This can be done by introducing the electrodynamic transduction efficiency $\eta_{e}$:

(53) $$\eta_{e}=\frac{{\bar{P}}_{e}}{{\bar{P}}_{j}}$$

Hence, finally, one can define the (global) efficiency of the actuator η as the product of $\eta_{k}$ by $\eta_{e}$

(54) $$\eta =\eta_{k}\eta_{e}=\frac{{\bar{P}}_{k}}{{\bar{P}}_{j}}$$

where the external Joule power supply ${\bar{P}}_{j}$ provided to the actuator is calculated as

(55) $${\bar{P}}_{j}=\frac{1}{T}\int_{0}^{T}VIdt$$

with $V=V_{ac}\sin\omega t$ being the applied voltage and *I* the electric current flowing through the piezo-electric element.

The rate of ${\bar{P}}_{j}$ not turned into ${\bar{P}}_{e}$ is converted into a variation of internal energy $\dot{Q}$ of the air inside the cavity (heat generation per unit time), which is transferred in part to the external ambient through a natural convection mechanism, and, in part, into enthalpy flow rate of the air leaving the orifice.

From the energy balance one obtains also:

(56) $$\eta_{e}=1-\frac{\dot{Q}}{{\bar{P}}_{j}}$$

Moreover, it is important to note that the energy conversion process from electrodynamic energy to flow kinetic energy depends intrinsically on the coupling degree between the two oscillators, i.e., the membrane and the Helmholtz one. From the dynamical point of view the coupling effect is represented by the internal pressure term acting as a forcing term on the Helmholtz oscillator; from the energy point of view, the coupling effect can be seen as a transfer of mechanical power (e.g, the work done by the internal pressure) to the Helmholtz oscillator, expressed by

(57) $${\bar{P}}_{mH}=\frac{1}{T}\int_{0}^{T}p_{i}A_{w}{\dot{x}}_{w}dt$$

It is clearly evident that, although the net mechanical power due to the pressure work introduced in Equation (50) is null, ${\bar{P}}_{mH}$ defined in the above equation is not. It is thus convenient to split ideally the conversion process from electrodynamic energy to flow kinetic energy into two steps: the conversion of electrodynamic energy $\bar{P_{e}}$ to mechanical power due to the pressure work (within the membrane oscillator), namely ${\bar{P}}_{mH}$, and the conversion of mechanical power to air flow kinetic energy (within the Helmholtz oscillator). In equation terms, once introduced the *internal* efficiencies:

(58) $$\eta^{*}=\frac{{\bar{P}}_{mH}}{{\bar{P}}_{e}}$$

(59) $$\eta^{**}=\frac{{\bar{P}}_{k}}{{\bar{P}}_{mH}}$$

the kinetic efficiency of Equation (52) can be interpreted as:

(60) $$\eta_{k}=\eta^{*}\eta^{**}$$

In discussing the results, it will be seen that $\eta^{**}$ is generally very high, and in fact, based on the considerations developed in Section 2.4, namely that the driving differential pressure and dynamic pressure at the orifice is of the same order of magnitude, it is expected to be of unit order. On the other hand, bearing in mind the last two terms at right hand side of Equation (50), one can easily predict that $\eta^{**}∼1/K=0.877$. The efficiency $\eta^{*}$ depends crucially on the coupling degree between the two oscillators, since it scales essentially as the ratio of diaphragm acoustic stiffness $k_{w}^{\prime }$ to structural diaphragm stiffness $k_{w}$ (whose expressions were given in Section 2.2); in other words, $\eta^{*}$ depends directly on CF. In the case of reduced coupling, $\eta^{*}$ is very low and it reduces dramatically also the kinetic efficiency of the device. Furthermore, note that a limited coupling degree does not imply a low air flow velocity; it means that a certain value of air jet velocity is obtained at the price of a very low energy efficiency.

Following Girfoglio et al. [39], the influence of the coupling degree of the oscillators on the kinetic energy efficiency will be hereafter discussed, by monitoring the internal efficiencies $\eta^{*}$ and $\eta^{**}$ defined by Equations (58) and (59). This will be made with the aid of Table 5 and Table 6, where the relevant efficiencies are reported as functions of the applied voltage, for the brass and aluminum actuators respectively, tested by de Luca et al. [32]. Note that for the first device the data refer to the modified resonance structural frequency (about 2200 Hz), whereas for the latter device the data refer to the modified resonance Helmholtz frequency (about 900 Hz).

As anticipated before, it is evident that, while $\eta^{**}$ is generally very high for both the devices, $\eta^{*}$ depends crucially on the coupling degree between the two oscillators. In the case of brass shim device having CF = 0.06 the amount of mechanical power transferred from the diaphragm oscillator to the Helmholtz one is remarkably reduced, hence $\eta^{*}$ is very low. Of course this reduces dramatically also the kinetic efficiency of the device and finally the global efficiency. For the other one the coupling parameter is CF = 1.88 and the actuator exhibits significantly higher values of $\eta^{*}$.

Finally, it should be observed that a low value of kinetic efficiency does not corresponds necessarily to a low value of air jet velocity at exit orifice, but it means that a certain air jet is achieved by a low energy conversion efficiency.

Girfoglio et al. [39] further noted that, for two cavities configurations which share the same diaphragm (introduced by Luo et al. [29]), computations show that for high coupling degree (which is the case of aluminum actuator discussed in the present paper) the global kinetic efficiency remains almost the same as compared to the base single cavity configuration, and therefore the kinetic efficiency of the single cavity is halved.

## 6. Conclusions

This paper aimed to give a comprehensive review of the state of the art of the capabilities of Lumped Element Modeling (LEM) of predicting the frequency response of piezo-driven synthetic jet devices, commonly used to control fluid flows (e.g., boundary layers, wakes, jets).

LEM is discussed in both the fluidic/acoustic and electro/acoustic domains, basically with reference to previous literature contributions. Nevertheless, some novel original parts have been developed, mainly concerned with the transfer from the mechanical to the electrical domain, and the determination of stationary-periodic analytical solutions for the jet velocity, as functions of the major mechanical and geometrical quantities, as well as of the operating conditions. Such analytical solutions have clearly evidenced the magnification factor of the actual device response with respect to the so-defined *incompressible* behavior for resonance operating frequencies. A complete dimensionless analysis has been reported, leading to the nondimensional forms of the governing equations which, as is well known, are related to the behavior of a two coupled mechanical and acoustic oscillators, the diaphragm and the Helmholtz one. Overall, the dimensionless analysis along with the analytical formulas describing the stationary-periodic jet response, allowed the recognition of the set of dimensionless parameters governing the operation of the device.

A second part of the paper has been devoted to the analysis of the device performances, going from the device frequency response essentially in terms of jet velocity to the estimate of the energy efficiency. For all the cases here considered, for the aim of providing useful and practical design and manufacturing information, specific attention has been paid to emphasize the role played by the nondimensional governing parameters, in particular the degree of coupling of the two oscillators.

As far as future LEM applications are concerned, very interesting trends are towards optimized design problems in order to maximize a target objective function, which can be conveniently selected among various integral quantities, e.g., volumetric flux, momentum flux, energetic efficiency, as was recently pointed out by Kordík and Trávníček [45]. A quite new research line is related to the development of Low Order Modeling (LOM), i.e., approximate models for fluid flows, which provide insight about the fundamental mechanisms featuring a particular flow, as described by Rowley and Dawson [46] who make use of both proper orthogonal decomposition (POD), as well as more recent techniques, such as the dynamic mode decomposition (DMD). Such a LOM model is particularly important if used in combination with flow control actuators (namely the SJ devices described in this paper) to alter the flow in some way. LOM could be coupled with well-developed LEM tools within a suited new LOM-LEM strategy.

## Figures and Tables

**Table 1:** Typical effort and flow variables.

Energy Domain	Effort, e(t)	Flow, f(t)
Mechanical	Force, *F*	Velocity, *U*
Mechanical	Pressure, $p_{i}$	Volumetric flow rate, *Q*
Electrical	Voltage, $V_{ac}$	Current, *I*

**Table 2:** Basic dimensionless variables of the problem.

$Re=\frac{\bar{U}d_{o}}{\nu }$	$S=\frac{\omega d_{o}^{2}}{\nu }$	$\frac{d_{o}}{d_{w}}$	$\frac{H}{d_{o}}$	$\frac{l_{e}}{H}$	$\frac{m_{c}}{m_{wt}}$	$CF=\frac{\omega_{wc}^{2}}{\omega_{w}^{2}}$	$St=\frac{\omega H}{c}$

**Table 3:** Features of the devices studied by Gallas et al. [23].

	Property	Case 1	Case 2
Geometry	Shim diameter (mm)	23	37
	Shim thickness (mm)	0.15	0.10
	Piezo-electric diameter (mm)	20	25
	Piezo-electric thickness (mm)	0.08	0.11
	Cavity diameter (mm)	23	37
	Cavity height (mm)	5.76	4.65
	Orifice diameter (mm)	1.65	0.84
	Orifice length (mm)	1.65	0.84
	$H/d_{o}$	3.5	5.5
	$l_{e}/d_{o}$	1	1
Shim (brass)	Young’s Module (Pa)	$8.963\times 10^{10}$	$8.963\times 10^{10}$
	Poisson’s Module	0.324	0.324
	Density (Kg/m^3^)	8700	8700
Piezo-electric	Young’s Module (Pa)	$6.3\times 10^{10}$	$6.3\times 10^{10}$
	Poisson’s Module	0.31	0.31
	Density (Kg/m^3^)	7700	7700
Frequency response	$f_{w}$ (Hz)	2114	632
	$f_{1}$ (Hz)	2167	324
	$f_{H}$ (Hz)	941	452
	$f_{2}$ (Hz)	918	880
	CF	0.07	2.85

**Table 4:** Features of the devices studied by de Luca et al. [33].

	Property	Brass	Aluminum
Geometry	Shim diameter (mm)	41	80
	Shim thickness (mm)	0.4	0.25
	Piezo-electric diameter (mm)	31.8	63.5
	Piezo-electric thickness (mm)	0.191	0.191
	Cavity diameter (mm)	41	80
	Cavity height (mm)	1.5	4
	Orifice diameter (mm)	2	5
	Orifice length (mm)	2	2
	$H/d_{o}$	0.75	0.8
	$l_{e}/d_{o}$	1	0.4
Shim	Young’s Module (Pa)	$9.7\times 10^{10}$	$7.31\times 10^{10}$
	Poisson’s Module	0.36	0.31
	Density (Kg/m^3^)	8490	2780
	Diaphragm damping ratio	0.03	0.03
Piezo-electric	Young’s Module (Pa)	$6.6\times 10^{10}$	$6.6\times 10^{10}$
	Poisson’s Module	0.31	0.31
	Density (Kg/m^3^)	8700	7800	
Frequency response	$f_{w}$ (Hz)	1667	432	
	$f_{1}$ (Hz)	1877	344
	$f_{h}$ (Hz)	1004	752
	$f_{2}$ (Hz)	891	945
	CF	0.19	1.38

**Table 5:** Efficiencies of brass actuator at modified resonance structural frequency. CF = 0.06. Reprinted with permission from [39].

$V_{\mathrm{ac}}$ (V)	$\eta_{k}$ (%)	$\eta^{**}$ (%)	$\eta^{*}$ (%)
25	4.7	85.5	5.5
30	5.3	85.3	6.3
35	5.9	85	6.9
40	6.4	84.8	7.5
45	6.8	85.5	7.9
50	7.3	88.6	8.2
55	7.5	86.6	8.7
60	7.6	85.6	8.9
65	7.9	87.1	9.1
70	8.1	87.8	9.2
75	8.5	83.7	10.1

**Table 6:** Efficiencies of aluminum actuator at modified resonance Helmholtz frequency. CF = 1.88. Reprinted with permission from [39].

$V_{\mathrm{ac}}$ (V)	$\eta_{k}$ (%)	$\eta^{**}$ (%)	$\eta^{*}$ (%)
25	79.2	86.6	91.6
30	79.5	87.6	90.7
35	79.7	88.2	90.4
40	80.7	86.4	93.4
45	80.3	87.7	91.6
50	80.2	87.9	91.3
55	82.3	82.3	88.9
60	81.8	87.5	93.4
65	81.8	87.8	92.1
70	80.1	87.3	91.8
75	81	87.9	92.2
